# Model-based clustering of time-dependent observations with common structural changes

**DOI:** 10.1007/s11222-025-10756-x

**Published:** 2025-10-28

**Authors:** Riccardo Corradin, Luca Danese, Wasiur R. KhudaBukhsh, Andrea Ongaro

**Affiliations:** 1 Department of Economics, Management and Statistics, University of Milano-Bicocca, Milano, 20136 Italy; 2 School of Mathematical Sciences, University of Nottingham, Nottingham, NG7 2RD United Kingdom

**Keywords:** Change points, COVID-19, Model-based clustering, Time series

## Abstract

We propose a novel model-based clustering approach for samples of time series. We assume as a unique commonality that two observations belong to the same group if structural changes in their behaviors happen at the same time. We resort to a latent representation of structural changes in each time series, based on random orders, to induce ties among different observations. Such an approach results in a general modeling strategy and can be combined with many time-dependent models already known in the literature. Our studies have been motivated by an epidemiological problem. Specifically, we want to provide clusters of different countries of the European Union where two countries belong to the same cluster if the spreading processes of the COVID-19 virus show structural changes at the same time.

**Supplementary Information:**

The online version contains supplementary material available at 10.1007/s11222-025-10756-x.

## Introduction

Detecting patterns and similarities between observations is a key topic in modern statistics, especially in large datasets from which we can extract information. Among fundamental approaches, cluster analysis comprises a collection of statistical tools to gather insights from a set of data by looking for groups with similar features, defining some homogeneous subsets of observations. In particular, in a model-based clustering setting, data are described by a mixture model where two observations belong to the same cluster if they are assigned to the same component of the mixture. Equivalently, each observed datum is associated with a latent parameter, which indexes a specific mixture component, and two observations are clustered together if their latent parameters have the same value. Here we consider a scenario involving multiple time series, with each observation representing a series of values observed over time. We introduce a new method for clustering these time series based on specific characteristics, such as shared structural changes over time.

While various methods exist for modeling time-dependent data, a desirable property is the ability to account for shocks in the observed values. These alterations occur when an external event significantly impacts the phenomena being analyzed, changing its future behavior. The identification of these points, and the study of models accommodating those structural changes, is usually termed *change points detection*. Seminal contributions on this topic are Page (1954, 1957), who employed a frequentist approach using hypothesis tests to identify a single structural change in a time-dependent parameter. Later, such approaches have been extended in different directions by considering different methods to detect a change point. These methods include parametric and nonparametric approaches and cases where the position of the change point is assumed to be known or unknown (see, e.g., Brodsky and Darkhovsky 1993). However, trying to detect a single change point might be too restrictive, since in many situations more than one change may occur. Different works then extended these methods to the problem of multiple change points detection. Niu et al. (2016) provided a general review of multiple change points detection methods for mean changes in Gaussian model. Inclan and Tiao (1994) proposed an iterative method to detect changes in the variance of a time series, where a change point is detected whenever an abrupt change occurs in the cumulative sum of squares of the time series realisations. Similarly,  Chen and Gupta (1997) proposed a method to infer the structural changes by selecting the configuration of change points that minimises the Schwarz information criterion. See Chen and Gupta (2012) for a review on models for change point models and possible extensions. Change points may also occur in different scenarios. For example, Young (2013) studied a mixture of regression models to estimate the distribution of the response variable, conditionally on the predictors, in the presence of change points in the regression effects.

Beside frequentist approaches, many contributions have been introduced also in the Bayesian literature. Early studies relied on hypothesis test construction to detect a single structural change, such as Chernoff and Zacks (1964). Recent contributions investigated different strategies to accommodate for multiple change points. Among these, Barry and Hartigan (1992, 1993) considered a formulation of the problem that is based on a Product Partition Model (PPM, Hartigan 1990) construction to detect mean changes in an observed time series, which is assumed to be Gaussian distributed. The underlying model is based on a latent random partition of the parameters, where two parameters belong to the same block of the partition if they share the same value, and two distinct blocks of the partition describe two different regimes of the observed quantities. Later this approach has been extended in many directions. Loschi and Cruz (2002) proposed a strategy to detect changes in both mean and variance of a Gaussian kernel distribution over time, based on the PPM construction. Quintana and Iglesias (2003) studied the connection between PPM and the Dirichlet process in the context of change points. Fuentes-García et al. (2010) proposed a similar approach, but considering as partition model the Exchangeable Partition Probability Function (EPPF) of a Dirichlet process and restricting the support of such EPPF to the random orders space by assigning probability zero to partitions that do not preserve the order. Later Martínez and Mena (2014) considered a transformation of the EPPF arising from a Pitman-Yor process (Pitman 1995) that preserves its properties, such as the symmetric structure of the corresponding probability distribution and the distribution of the number of distinct blocks, but restricts the support of the function to the space of random orders, following the pioneering studies of Pitman (2006). More recent contributions considered multivariate cases (Corradin et al. 2022), correlated change points for multiple time-dependent observations (Quinlan et al. 2024), different change points for different parameters (Pedroso et al. 2023) and models where change points detection is performed assuming a temporal dependency among random partitions (Page et al. 2022; Giampino et al. 2024). Further, under suitable assumptions of the local model, these combinatorial approaches can be seen as infinite hidden Markov models (Beal et al. 2001) with upper triangular transition matrix.

We introduce an approach that employs a model-based clustering framework to group observations exhibiting common structural changes, while other individual parameters and regimes are observation-specific. Our goal is to cluster observations that experience a shock simultaneously, even if their behaviors differ. To our knowledge, the use of latent combinatorial structures for model-based clustering of time series, that primarily focus on change points, has not been investigated yet. However, there are various contributions aimed at clustering multiple time series, possibly with regime changes. For a comprehensive review of different clustering techniques for time series see Aghabozorgi et al. (2015). In a Bayesian framework, early contributions to time series model-based clustering can be found in Frühwirth-Schnatter and Kaufmann (2008), who examined a scenario with a fixed number of clusters, grouping observations based on latent parameters that define their behaviors. Later extensions include different kernel functions (Juárez and Steel 2010) and categorical data (Frühwirth-Schnatter and Pamminger 2010), among others. Allied to our approach, (Same et al. (2011)) propose a model to cluster together time series with changes in their regime, by resorting to a polynomial representation of the time dependent observations, whose degree can be different over time. Further, Brault et al. (2024) introduce a method that clusters together functional data with structural changes. The latter not only clusters data with similar behavior, but also detects change points for the functions in each group. However, differently from our approach, here the clustering is focusing on similarities of local behaviors, rather than primarily on change points.

Our primary motivation behind this study comes from the recent COVID-19 pandemic. Epidemic models such as the Kermack–McKendrick renewal equation models (Kermack and McKendrick 1927) and their stochastic analogues have been studied for many decades now (Andersson and Britton 2000). Along with their study, parameter inference methods have been also developed (see, e.g, Niels 1993; Becker 1993; Choi and Rempala 2011; Raúl Fierro and Balakrishnan 2015; Kypraios et al. 2017). See Britton and Pardoux (2019) for an overview on this topic. Due to the recent COVID-19 pandemic, these methods received a renewed interest. For example, frequentist methods (Bong et al. 2024) and other techniques for calibration of computer models (Fadikar et al. 2018; Baker et al. 2022) have been investigated successfully in the last years. Similarly, the problem of parameter inference from a Bayesian perspective was discussed for mechanistic (Seymour et al. 2022; Chitwood et al. 2022; KhudaBukhsh et al. 2023) and semi-mechanistic models of COVID-19 (Bhatt et al. 2023). More recent contributions accommodate for different behaviour of the model over time. Specifically, Hong and Li (2020), Gleeson et al. (2021) and Wascher et al. (2024) discussed models with time-varying infection rates, while structural changes in such parameters were modeled in Huang et al. (2024). Here, we want to cluster different countries on the basis of their structural changes in the dynamics dictated by a stochastic epidemic model.

The paper is structured as follows. Section 2 introduces a model to cluster together time-dependent observations with common change points. Section 3 describes the distributions of interest that can be used to construct a sampling strategy. In Section 4 we present a Markov chain Monte Carlo (MCMC) procedure to sample realizations from the posterior distribution of interest. In Section 5 the properties of the model are studied with an application to synthetic and real time series data. Section 6 shows the epidemiological study which motivates our contribution. Conclusions and future developments are discussed in Section 7. Further details on algorithm implementations, illustrations, and the epidemiological model are deferred to the supplementary material. Finally, an implementation of the methodologies presented in this manuscript is available in the R package BayesChange (Danese et al. 2025).

## Modeling multiple time series with common change points

We introduce a novel approach to cluster together time series with respect to common structural changes, and no further local similarities are assumed among them. In this sense, observations might be locally indexed by different subject-specific behaviours, but cluster together if they change regime on the same time instants. Let $\boldsymbol{y}_i = \{y_{i,1}, \dots , y_{i,T}\}$, be the generic *i*th time series we observe, where $y_{i,t}$ is a random quantity taking values on a space $\mathbb Y$, which depends on the specific quantity we observe, and $\mathcal Y = \{\boldsymbol{y}_1, \dots , \boldsymbol{y}_n\}$ denotes the whole sample. For simplicity, here we assume that time series are observed at the same discrete times $\{1, \dots , T\}$, however our approach works even in more general scenarios. See, e.g., Section 6 where we consider a model tailored for epidemiological spreads as kernel function.

Regarding the distribution of each time series, we assume that our data are sharing the same model structure over time with Markovian dependence, where $\mathcal {L}(y_{i,t} \mid y_{i,t-1}, \theta _{i,t}), \, t = 1, \dots , T$ denotes the distributional law of $y_{i,t}$ given the previous observation $y_{i,t-1}$ and the local parameter $\theta _{i,t}$, with $\theta _{i,t} \in \Theta$ for all $i = 1, \dots , n$ and $t = 1, \dots , T$. We remark that the modeling structure presented in the manuscript can be extended to cases with longer memories by substituting the distribution $\mathcal {L}$ with suitable alternatives. The local behaviour of the model for the *i*th observation is dictated by the sequence of latent parameters $\theta _{i,1}, \dots , \theta _{i,T}$, which are assumed to have ties over time. A change point for the *i*th time series occurs when the parameter at time *t* differs from the previous value, i.e. $\theta _{i,t} \ne \theta _{i,t-1}$. Equivalently, each observation is divided into one or more blocks, where realisations within each block share the same value of $\theta _{i,t}$. When the value of the parameter changes a change point occurs and a new block begins. The distribution of the sequence of parameters $\theta _{i,1}, \dots , \theta _{i, T}$ can be factorised into two independent terms

1 $$\begin{aligned} \mathcal {L}(\theta _{i,1}, \dots , \theta _{i,T}) = \mathcal {L}(\rho _i) \mathcal {L}(\theta _{i,1}^*, \dots , \theta _{i,m_i}^*). \end{aligned}$$

The first term $\mathcal {L}(\rho _i)$ denotes the distribution of the random order $\rho _i$ inducing changes in the sequence of parameters $\theta _{i,1}, \dots , \theta _{i,T}$. Here, by random order $\rho _i$ we mean a random partitioning of $\{1, \dots , T\}$ into $m_i$ blocks $A_{i,1}, \dots , A_{i, m_i}$ satisfying the following properties:

- $A_{i,\ell } \cap A_{i, j} = \emptyset$ for all $\ell , j \in \{1, \dots , m_i\}$ with $\ell \ne j$;
- $$A_{i,1} \cup \cdots \cup A_{i,m_i} = \{1, \dots , T\}$$;
- For any $j, \ell \in \{1, \dots , m_i\}$ with $j < \ell$, $w \in A_{i,j}$ and $s \in A_{i,\ell }$, we have $w < s$.

The first two conditions are properties of a generic partition of *T* elements, while the latter restricts the partition space to the subset satisfying the ordering constraint. The second term in (1) denotes the distribution of $\theta _{i,1}^*, \dots , \theta _{i,m_i}^*$, the unique values out of $\theta _{i,1}, \dots , \theta _{i,T}$, whereas the generic $\theta _{i,j}^*$ determines a common local behaviour and it is shared by all the observations whose times are allocated in the *j*th block of $\rho _i$. Hence, a time series switches its regime when it moves from a block to another of $\rho _i = \{ A_{i,1}, \dots , A_{i, m_i}\}$, while the local behaviours are fully characterised by $\theta _{i,1}^*, \dots , \theta _{i,m_i}^*$.

**Fig. 1:**
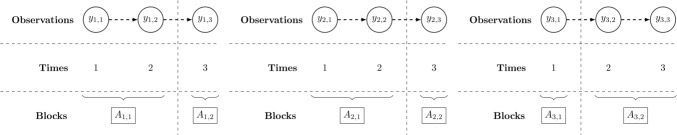
Graphical representation of three time series $\boldsymbol{y}_1, \boldsymbol{y}_2, \boldsymbol{y}_3$, where each series is observed at $T=3$ times, with their corresponding latent partition, whereas $\boldsymbol{y}_1$ and $\boldsymbol{y}_2$ are in the same cluster while $\boldsymbol{y}_3$ is in a separate cluster

Our aim is to cluster time series in homogeneous groups sharing the same change points. To this end, we assume the latent orders $\mathcal R = \{\rho _1, \dots , \rho _n\}$ distributed independently according to a discrete distribution $\tilde{p}$, with

2 $$\begin{aligned} \tilde{p}(\rho ) = \sum _{r=1}^{2^{T-1}}\pi _r\delta _{ \tilde{\rho }_r}(\rho ), \end{aligned}$$

where $\{\tilde{\rho }_1, \dots , \tilde{\rho }_{2^{T-1}}\}$ is an exhaustive collection of all the possible orders of *T* elements, $(\pi _1, \dots , \pi _{2^{T-1}})$ is a vector of probabilities taking values in the $(2^{T-1}-1)$-dimensional simplex space, and $\delta _a(\cdot )$ denotes a Dirac measure at *a*. The discreteness of (2) implies a positive probability of having ties in $\mathcal R$. Ties represent clusters of observations sharing the same change points, i.e. changing their local behaviour at the same time. The observations are then divided according to a partition $\lambda =\{ B_1, \dots , B_k\}$ of the indices $\{1, \dots , n\}$, for which $B_\ell \cap B_j = \emptyset$ for all $\ell , j \in \{1, \dots , k\}$ with $\ell \ne j$, and $B_1 \cup \cdots \cup B_k = \{1, \dots , n\}$. Figure 1 shows an example with $n = 3$ time series observed for $T = 3$ time instants with a single change point. The left and middle series belong to the same cluster, as the change point occurs at $t=3$, while the right one forms a stand-alone cluster with a change point at time $t = 2$.

The model specification is completed by setting a distribution for the weights $\pi _1, \dots , \pi _{2^{T-1}}$, in the following sections assumed to be Dirichlet distributed, by choosing a distribution for the observed data $\mathcal {L}(y_{i,t}\mid y_{i,t-1}, \theta _{i,t})$ and by setting a prior distribution for the parameters $\theta _{i,t}$s, here denoted by $P_0$. We remark that, since each observed time series is associated with a latent order, its likelihood contribution can be factorised as a product of terms sharing the same local behaviour. The model we consider can be then written in its hierarchical form as

3 $$\begin{aligned} \begin{aligned}&\boldsymbol{y}_i \mid \rho _i, \boldsymbol{\theta }_i^* \sim \prod _{j=1}^{m_i} \prod _{t = t_{i,j}^-}^{t_{i,j}^+} \mathcal {L}(y_{i,t}\mid y_{i,t-1}, \theta _{i,j}^*),\quad i = 1, \dots , n,\\&\rho _i \mid \tilde{p}(\rho ){\mathop {\sim }\limits ^{{\text{ iid }}}}\tilde{p}(\rho ), \quad i = 1, \dots , n,\\&(\pi _1,\dots ,\pi _{2^{T-1}}) \sim \textsc {Dir}(\alpha _1, \dots , \alpha _{2^{T-1}}),\\&\theta _{i,j}^*{\mathop {\sim }\limits ^{{\text{ iid }}}}P_0(\theta ),\quad j = 1, \dots , m_i, \; i = 1, \dots , n, \end{aligned} \end{aligned}$$

where $t_{i,j}^- = \min \left\{ t: t \in A_{i,j} \right\}$ denotes the first time index belonging the *j*th block of the latent order associated with the *i*th observation, $t_{i,j}^+ = \max \left\{ t: t \in A_{i,j} \right\}$ the last time index, and with the proviso that the first time of a new regime in the observed quantities does not depend on observed values in past regimes, i.e. $\mathcal {L}(y_{i,t_{i,j}^-} \mid y_{i,t_{i,j}^- - 1}, \theta _{i,j}^*) = \mathcal {L}(y_{i,t_{i,j}^-} \mid \theta _{i,j}^*)$.

Specific distributional choices of $\mathcal {L}(y_{i,t}\mid y_{i,t-1},\theta _{i,t})$ depend on the data we are considering. For example, in the following sections we present an illustration where the data are real-valued quantities modeled through an univariate Ornstein-Uhlenbeck process (Section 5) and a case with survival data arising from a susceptible-infected-removed model (Section 6). However, any time-dependent model with discrete or discretisable observational time can be embedded in the specification of a model as in (3). The distribution of the parameters $\mathcal T^* = \{\boldsymbol{\theta }_1^*, \dots , \boldsymbol{\theta }_n^*\}$ depends on the distributional assumption of $\mathcal {L}(y_{i,t}\mid y_{i,t-1},\theta _{i,t})$. Futher, $\mathcal T^*$ can be marginalised in case we are not interested on posterior inference for local behaviours, but only on common structural change times.

## Posterior distribution of interest

Our main interest lies in the latent partition defining groups of time series which share the same change points. The posterior distribution of such quantity is not available in a closed form, but we can identify a tractable expression from which we can produce a sample. Hence, we start from the joint distribution of the observed time series $\mathcal Y$, latent orders $\mathcal R$, unique values of the parameters $\mathcal T^*$ and the vector of probabilities $\boldsymbol{\pi }= (\pi _1, \dots , \pi _{2^{T-1}})$. The distribution of the previous quantities can be then expressed, in force of the chain rule, as product of the corresponding conditional distributions, with

4 $$\begin{aligned} \mathcal {L}(\mathcal Y,&\mathcal T^*, \mathcal R, \boldsymbol{\pi }) \nonumber \\ &= \mathcal {L}(\mathcal Y \mid \mathcal T^*, \mathcal R) \mathcal {L}(\mathcal T^* \mid \mathcal R) \mathcal {L}(\mathcal R \mid \boldsymbol{\pi }) \mathcal {L}(\boldsymbol{\pi }), \end{aligned}$$

where $\mathcal Y$ is independent of $\boldsymbol{\pi }$ conditioned on $\mathcal T^*$, $\mathcal R$ and $\mathcal T^*$ is also independent of $\boldsymbol{\pi }$ conditioned on $\mathcal R$. We can make the joint distribution in (4) explicit by using the distributions in (3), with the likelihood term, here denoted by $\mathcal {L}(\mathcal Y \mid \mathcal T^*, \mathcal R)$, being the product of the data distribution for all the observed time series. We then have

5 $$\begin{aligned} \mathcal {L}(\mathcal Y, \mathcal T^*,&\mathcal R, \boldsymbol{\pi })= \prod _{i=1}^n\prod _{j=1}^{m_i} \prod _{t = t_{i,j}^-}^{t_{i,j}^+} \mathcal {L}(y_{i,t}\mid y_{i,t-1}, \theta _{i,j}^*)\nonumber \\ &\times \prod _{i=1}^n \prod _{j=1}^{m_i} P_0(\theta _{i,j}^*) \prod _{i=1}^n \tilde{p} (\rho _i) \frac{1}{\mathrm B(\boldsymbol{\alpha })}\prod _{r = 1}^{2^{T-1}} \pi _r^{\alpha _r - 1},\nonumber \\ \end{aligned}$$

where $B(\boldsymbol{\alpha })$ denotes the multivariate beta function of parameter $\boldsymbol{\alpha }$.

We are interested in partitioning the observations on the base of their latent orders. Therefore, the unique values of the parameters $\mathcal T^*$ can be marginalised from the previous equation, obtaining the following expression

$$\begin{aligned} \mathcal {L}(\mathcal Y, \mathcal R, \boldsymbol{\pi })=&\prod _{i=1}^n\prod _{j=1}^{m_i}\mathcal M(\{y_{i,t} \mid t \in A_{i,j}\}) \\ &\times \prod _{i=1}^n \sum _{r=1}^{2^{T-1}} \pi _r\delta _{\tilde{\rho }_{r}}(\rho _i) \frac{1}{\mathrm B(\boldsymbol{\alpha })}\prod _{r = 1}^{2^{T-1}} \pi _r^{\alpha _r - 1}, \end{aligned}$$

whereas $\mathcal M(\{y_{i,t} \mid t \in A_{i,j}\})$ denotes the marginal distribution of the *i*th time series in the *j*th block, resulting from integrating the local parameter $\theta _{i,j}^*$, i.e.

6 $$\begin{aligned}&\mathcal M(\{y_{i,t} \mid t \in A_{i,j}\}) \nonumber \\ &\qquad = \int \prod _{t = t_{i,j}^-}^{t_{i,j}^+} \mathcal {L}(y_{i,t}\mid y_{i,t-1}, \theta _{i,j}^*) P_0(\theta _{i,j}^*) \text {d}\theta _{i,j}^*. \end{aligned}$$

Clearly, the tractability of (6) depends on specific choices of the data distribution $\mathcal {L}(y\mid \cdots )$ and the prior for the local parameter $P_0(\theta _{i,j}^*)$. In some common scenarios, the integral can be solved analytically, obtaining a closed form expression for the marginal distribution, as done for example in Section 5. Alternatively, for more complex scenarios such integral can be approximated through numerical solutions. See, e.g., Section 6.

Finally, we can marginalise also the weights $\boldsymbol{\pi }$, as we are not interested on estimates of the mixing distribution but on the clustering, obtaining the following distribution

7 $$\begin{aligned} \mathcal {L}(\mathcal Y, \mathcal R) =&\frac{\Gamma (\alpha ^+)}{\Gamma (\alpha ^+ + n)}\prod _{r = 1}^{2^{T-1}} \frac{\Gamma (\alpha _r + n_r)}{\Gamma (\alpha _r)} \nonumber \\ &\times \prod _{\{i: \rho _i = \tilde{\rho }_r\}}\prod _{j=1}^{m_i}\mathcal M(\{y_{i,t} \mid t \in A_{i,j}\}), \end{aligned}$$

where $\alpha ^+ = \sum _{r=1}^{2^{T-1}} \alpha _r$, $\{i:\rho _i = \tilde{\rho }_r\}$ denotes the set of indices for which the latent order $\rho _i$ coincides with the *r*th element of $\tilde{p}$, and $n_r$ is the cardinality of $\{i:\rho _i = \tilde{\rho }_r\}$, i.e., number of observations associated with the cluster with latent order $\tilde{\rho }_r$. A model of the form in (7) describes the convolution of a Dirichlet-categorical model with the marginal distributions of the data. We further remark that the partition of the data $\lambda$ is then given by the equivalence classes described by allocating observations to different latent orders, with $\lambda = \{B_1, \dots , B_k\}$ whereas $B_j = \{ i: \rho _i = \rho _{(j)}^\dagger \}$ is the *j*th block of the partitions whose latent orders are equal to $\rho _{(j)}^\dagger$, and $\{\rho _{(1)}^\dagger , \dots , \rho _{(k)}^\dagger \}$ denotes the set of orders out of $\tilde{\rho }_1, \dots , \tilde{\rho }_{2^{T-1}}$ with at least one observation assigned, i.e. the elements in $\{\rho _r: n_r > 0\}$. In the following, the model is elicited by considering an objective prior specification for the weights, i.e., $\alpha _r = \alpha$ for all $r = 1, \dots , 2^{T-1}$. We recall that the probability of having ties while sampling two observations from a Dirichlet-categorical model is given by

$$\begin{aligned} P(X_2 = X_1) = \frac{\alpha + 1}{2^{T-1} \alpha + 1} \end{aligned}$$

with $\lim _{\alpha \rightarrow 0} P(X_2 = X_1) = 1$ and $\lim _{\alpha \rightarrow +\infty } P(X_2 = X_1) = \frac{1}{2^{T-1}}$. Therefore, to ensure a significant probability of a tie when dealing with large values of *T*, in the following we set $\alpha = 1$.

## Informed split-and-merge algorithm

The model we consider in the manuscript has a flexible structure which can accommodate for various types of data. The bottleneck of facing posterior inference is due to two main sources: the distribution we assume for the data $\mathcal {L}(y_{i,t}\mid y_{i,t-1}, \theta _{i,t})$, which depends on specific analyses we are performing, and the cardinality of $\tilde{p}$. Regarding the latter, the number of latent orders scales poorly with the number of observed values for the time series, with $2^{T-1}$ possible orders when we observe *T* distinct times. Even for a small *T*, we cannot explore the whole partitions’ space in a reasonable time, and we need to use suitable computational strategies to obtain a sample from the target distribution of interest.

Among the possible approaches, we build a sampling strategy upon the split-merge algorithm for nonparametric priors and clustering problems (Green and Richardson 2001; Jain and Neal 2004), a particular type of MCMC algorithm that can be used to update the latent partition $\lambda$ of a model as in (3). Intuitively, at each step of the algorithm two indices of the latent partition, $i,\ell \in \{1, \dots , n\}$ with $i \ne \ell$, are randomly chosen. If the two indices belong to the same block of $\lambda$, meaning that the *i*th and the $\ell$th observations belong to the same cluster, their cluster is randomly split in two blocks. Otherwise, if the two indices *i* and $\ell$ belong to different blocks of $\lambda$, the two clusters are merged. Once the split or the merge step is completed, we obtain a candidate partition $\lambda ^{(N)}$ to update the current state of chain. We then perform a Metropolis–Hastings step to accept $\lambda ^{(N)}$ or to stay on the previous state of the chain. For the sake of clarity, we report in Algorithm 1 the pseudo-code to sample *M* values from the posterior distribution of the latent partition $\lambda$.

**Algorithm 1:**
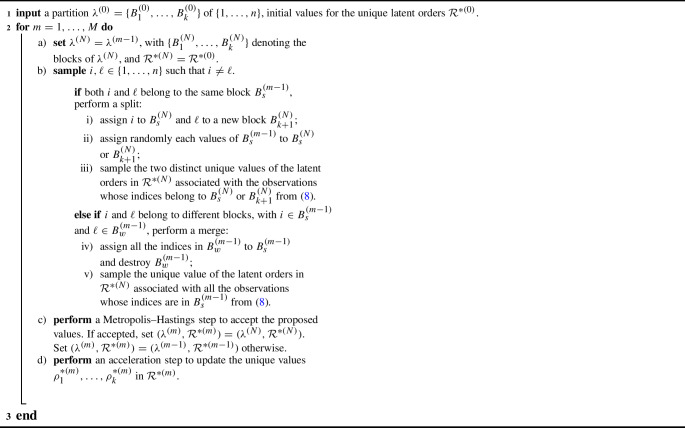
Split-merge algorithm to sample from $\mathcal {L}(\lambda \mid \mathcal Y)$

The main challenge of using the split-merge algorithm in our framework is the sparse nature of $\tilde{p}$. When we perform a split or merge step, we need to propose a new latent order for each new block we create, as in step iii) and v) of Algorithm 1. Because $\tilde{p}$ has a large cardinality, randomly proposing those orders from a distribution independent of the data or previous state makes them unlikely to be representative of the new clusters, even when the proposed partition $\lambda ^{(N)}$ is a suitable candidate. To avoid this problem, we resort to an informed proposal for those orders. In particular, inspired by Zhang et al. (2022), we included in the proposal specification the information arising from the observed time series. Hence, our proposal equals

8 $$\begin{aligned} \psi (\rho \mid \mathcal Y) = \sum _{i=1}^n \frac{1}{n} \mathcal {L}(\rho \mid \boldsymbol{y}_i), \end{aligned}$$

i.e. a mixture of the posterior distribution of the latent orders conditionally on each observation separately. Here, $\mathcal {L}(\rho \mid \boldsymbol{y}_i)$ denotes the posterior distribution of the latent order $\rho$ given the *i*th time series $\boldsymbol{y}_i$. Such mixture strategy for the proposal shrinks the probability mass across the space of orders $\rho _1, \dots , \rho _{2^{T-1}}$ to the elements which are likely at least for a single observations, decreasing the probability of sampling values which are not representative of any observed time series. While sampling from a generic component of (8) is feasible, evaluating the entire mixture requires the computation of *n* normalization constants, intractable for large *T*. These normalization constants can be computed before starting the algorithm. For example, we considered an importance sampling strategy with uniform importance distribution over the partitions’ space (see Section C of the supplementary material). Further details on the implementation are deferred to Section A of the supplementary material.

## Real-valued time series clustering

Before extending our model strategy to more complicated scenarios, we first investigate the performance of Algorithm 1 with a synthetic study on time series and then show an application on a real financial scenario. We consider a case where data are real-valued time series, for which structural changes occur at certain times. The generic *j*th block of the *i*th time series is generated according to the following process

9 $$\begin{aligned} y_{i,t} = {\left\{ \begin{array}{ll} \mu _{i,t} + N(0,\eta _{i,t}), & \text{ if } t = 1,\\ \gamma \, y_{i,t-1} + (1 - \gamma ) \, \mu _{i,t} + N\left( 0, (1 - \gamma ^2) \eta _{i,t}\right) , & \text{ otherwise, } \end{array}\right. } \end{aligned}$$

where *N*(*a*, *b*) denotes the Gaussian distribution with expectation *a* and variance *b*. We consider a scenario with three groups of time series. Each series is observed $T = 300$ times divided into $k_i$ distinct regimes, $i=1, \dots , 10$, and series belonging to the same cluster have change points at the same time but different local parameters. For all the time series, we fix $\gamma = 0.1$. We set the local unique values of the parameter, i.e. block-specific values $\mu _{i,j}^*$s out of $\mu _{i,1}, \dots , \mu _{i,T}$ and $\eta _{i,j}^*$s out of $\eta _{i,1}, \dots , \eta _{i,T}$ according to the scheme in Table 1, so that change points occur at different times in different clusters. An example of the sampled data are shown in Figure 1 of the supplementary material.

**Table 1:** Data generating process parameters for the the synthetic study with real-valued time series. Left to right: observation index, local trend parameters, local dispersion parameters, and true latent order shared among observations in the same cluster

*i*	$\{\mu _{i,1}^*, \dots , \mu _{i,k_i}^*\}$	$\{\eta _{i,1}^*, \dots , \eta _{i,k_i}^*\}$	$\{|A_{i,1}|, \dots , |A_{i,k_i}|\}$
1	{-0.25,-0.5,0.5,0,0.5}	{0.1,0.12,0.14,0.1,0.13}	{50,100,45,55,50}
2	{0.1,0.12,0.14,0.1,0.13}	{0.1,0.12,0.14,0.1,0.13}	
3	{0.45,-0.5,0.5,0,0.5}	{0.1,0.12,0.2,0.12,0.14}	
4	{0.5,-0.5,0.5,0.5,0}	{0.1,0.12,0.09,0.24,0.15}	
5	{-0.5,0,1,-0.1,0.6,-0.2}	{0.14,0.13,0.17,0.12,0.14,0.12}	{40,50,45,45,30,90}
6	{0,0.85,-0.15,0.65,0,1}	{0.1,0.24,0.14,0.15,0.12,0.13}	
7	{0.4,0.4,-0.15,0.5,0,-0.65}	{0.22,0.12,0.1,0.13,0.14,0.12}	
8	{-0.5,0,1,-0.1,0.6,-0.2}	{0.14,0.13,0.17,0.12,0.14,0.12}	{75,50,40,20,75,40}
9	{0.5,0.5,1,0.25,-0.5,0.25}	{0.12,0.22,0.15,0.14,0.17,0.19}	
10	{-0.5,0,0.5,-0.1,0.6,-0.25}	{0.12,0.13,0.15,0.12,0.15,0.18}	

We analyze the data considering a model as in (3). In particular, we assume that within each block the data follow an univariate Ornstein-Uhlenbeck process (Uhlenbeck and Ornstein 1930). The process defined as the solution to the following stochastic differential equation

10 $$\begin{aligned} dY_t = -\alpha (Y_t - \mu )dt + \sqrt{\frac{2\alpha }{\eta }}dW_t, \end{aligned}$$

where $\mu \in \mathbb R$, $\alpha , \eta > 0$, $\{W_t\}_{t \ge 0}$ is the Wiener process, and $\alpha$ is the parameter tuning the dependence over time. This specific model choice, once integrated at discrete times, leads to a model of the form in Equation 9. Here, the generic parameter indexing locally the time series is given by $\theta _{i,t} = (\mu _{i,t}, \eta _{i,t})$, while the parameter $\alpha$ (or equivalently $\gamma = e^{-\alpha }$) is assumed to be fixed and shared for all the time series. By setting $\mu \mid \eta \sim \text {Normal}(0, (c\eta )^{-1})$ and $\eta \sim \text {Gamma}(a,b)$, Martínez and Mena (2014) have shown that the marginal distribution of the data becomes

11 $$\begin{aligned}&\mathcal {M}(\{y_{i,t}:t \in A_{i,j}\} \mid \gamma ) \nonumber \\&\quad = \frac{(2b(1-\gamma ^2))^a \Gamma (n_j/2 + a)}{\pi ^{n_j/2}\Gamma (a)}\left( \frac{c(1+\gamma )(1-\gamma ^2)}{c+n_j-\gamma (n_j-c-2)}\right) ^{1/2} \nonumber \\&\times \Bigg ( \boldsymbol{y}_j^{\intercal } \boldsymbol{S}_j \boldsymbol{y}_j - \frac{(1-\gamma ) (\sum _{i=1}^{n_j}y_{j,i} - \gamma \sum _{i=2}^{n_j-1}y_{j,i})^2}{c+n_j-\gamma (n_j-c-2)} \nonumber \\&+ 2b(1-\gamma ^2) \Bigg )^{-(n_j/2 + a)}, \end{aligned}$$

where $\boldsymbol{y}_j = \left( y_{i,t_{i,j}^-}, \dots , y_{i,t_{i,j}^+}\right)$ and $\boldsymbol{S}_j$ is an $n_j \times n_j$ matrix of the form

$$\begin{aligned} \boldsymbol{S}_j = \begin{bmatrix} 1 & -\gamma & 0 & \dots & 0\\ -\gamma & 1 + \gamma ^2 & -\gamma & \dots & 0 \\ 0 & -\gamma & 1 + \gamma ^2 & \dots & 0 \\ \vdots & \vdots & \vdots & \ddots & \vdots \\ 0 & 0 & 0 & \dots & 1 \end{bmatrix}. \end{aligned}$$

Posterior inference is performed exploiting Algorithm 1. We consider different scenarios by combining together different values of the parameters tuning such algorithm. Specifically, we vary the accuracy of the normalization constant in Equation 8 by considering different numbers of values in the importance sampling $\mathrm B \in \{1\,000, 10\,000, 100\,000\}$. Further, when we propose a candidate from Equation 8, we consider different depths of the proposal, i.e. the number of sampling steps $\mathrm L\in \{1, 25, 100\}$. To understand the impact of the latter, while proposing a candidate random order, we are randomly selecting one of the *n* dimensions in (8), then we sample from the corresponding posterior distribution. For the latter, we initialise randomly the latent order and we then perform *L* split-merge step (Martínez and Mena 2014) to update that value. Each scenario considered in the study is replicated 50 times.

The model specification is completed by specifying the parameters of the Dirichlet distribution in Equation 7, with $\alpha _r = \alpha = 1$ for every $r \in \{1,\dots ,2^{T-1}\}$. Following the approach by Martínez and Mena (2014), the likelihood of a time series $y_i$ is given by (11) where $\mu$ and $\eta$ have been marginalised after setting respectively a normal and a gamma prior distributions. According to the notation introduced beforehand, we set as parameters for the gamma $a = b = 1$ and $c=0.1$ for the normal distribution. Finally, we set the autocorrelation parameter $\gamma = 0.1$, matching the values used to simulate the data. For the split-merge procedures that compute the marginal change points we set the probability of performing a split to $q=0.5$.

**Table 2:** Posterior summaries of the time series simulation study. We consider different accuracies of the normalization constant $\mathrm B$ and different proposal depth $\mathrm L$. The table reports the Binder loss between the point estimate and the true latent partition of the data ($\textrm{BI}$). Results are averaged over 50 replicates

$\mathrm B$	$\mathrm L$	$\textrm{BI}(\hat{\lambda }, \lambda _0)$	$\mathrm B$	$\mathrm L$	${\textrm{BI}}(\hat{\lambda }, \lambda _0)$	$\mathrm B$	$\mathrm L$	${\textrm{BI}}(\hat{\lambda }, \lambda _0)$
$10^3$	1	0.251	$10^4$	1	0.270	$10^5$	1	0.282
	25	0.146		25	0.165		25	0.182
	100	0.025		100	0.051		100	0.078

We run each posterior sampling for $5\,000$ iterations of which $2\,000$ are discarded as burn-in period. To avoid label-switching problems, we obtain the point estimate of the latent partition $\hat{\lambda }$ as the one among those visited by the algorithm that minimizes the expected Binder loss function (Binder 1978), following a decisional approach early introduced by Lau and Green (2007) and later studied by Wade and Ghahramani (2018). Table 2 shows the Binder loss function measured among the true and the estimated partition of the data, averaged over the 50 replicates, hence measuring how close are our estimates to the partitions that actually generated the synthetic data. Algorithm 1 performs consistently well with all configurations. Both the accuracy of the normalization constants $\mathrm B$ and the proposal depth $\mathrm L$ seem to affect systematically its performance. Intuitively, large $\mathrm B$ and $\mathrm L$ should be preferred, as they increase the precision of the proposal and of its sampling step. However, small values of $\mathrm L$ strongly reduce the computational time needed to perform the sampling. Results also suggest that a large value of *B* is not needed, however if the number of realizations increases we suggest to increase also *B*.

**Fig. 2:**
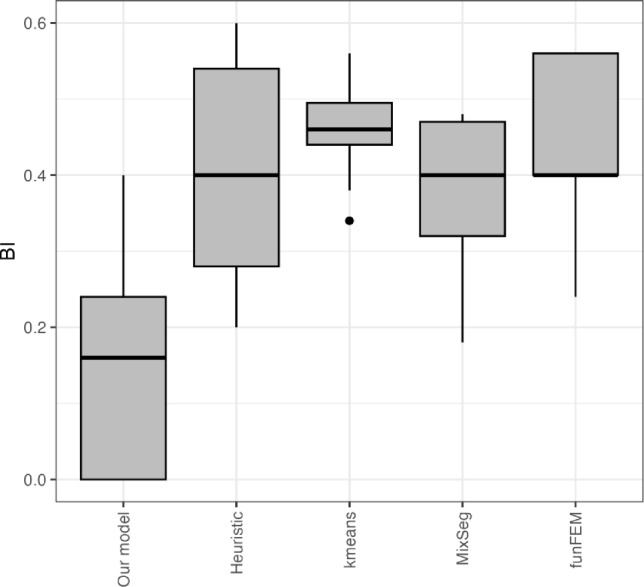
Binder Loss (BI) distance between the estimated partition and the true partition over 50 replications of simulated data. Left to right: our proposal, an heuristic approach based on marginal estimates of change points, a distance-based approach among time series, the MixSeg model and the funFEM model

We compare the performance of our model with competitor approaches, in terms of partition estimates. Regarding our proposal, we kept the previous specification of the model’s parameters, with $B = 1\,000$ and $L = 25$, balancing computational time and accuracy. The first competitor we consider is an heuristic procedure, where we first estimate the change points marginally for each time series and then we group the observations resorting to a hierarchical clustering algorithm. The second competitor works in the spirit of k-means algorithms, looking at the dynamic time warping among different time series, here treated as functional observations. The third one is the *MixSeg* algorithm by Brault et al. (2024). Finally the *funFEM* algorithm by Bouveyron et al. (2015). We run each model on 50 replications of simulated data from the same study in Table 1. Similarly to the previous synthetic study, for each run and each model we evaluated the Binder loss function between the true and the estimated partitions. Small values mean that the estimated partition is close to the true one. As shown in Figure 2, our model performs better than the others, producing more accurate estimates of the data clustering structure. Nevertheless, as for most competitors considered in the study, the accuracy of estimating the latent partition involves a moderate level of uncertainty, as evidenced by the dispersion observed in the boxplots of Figure 2.

### Change point in currency rates

After presenting the properties of the model on synthetic time series, we provide an application to financial data. Specifically we consider the time series of the daily Euro exchange rate to the other main currencies^1^. An exchange rate is the value at which one currency can be exchanged for another one, its evolution is strongly determined by the economy of the countries that adopt that currency. We analyze the period that goes from 1st January 2022 to the 31st of January 2024, the total amount of time observations is 768 and the number of exchange rates is 29. We excluded the Russian Ruble, for which we are missing data for a long period of time, and the Bulgarian Lev. Time series have been marginally standardized before running the analysis. Further, we complete the model specification by setting $a = b = 1$, $c=0.1$, and $\alpha = 1$. The autocorrelation parameter is also fixed $\gamma = 0.1$, however we perform a sensitivity analysis without any evidence of changing in the results while varying $\gamma$, except for large values introducing a strong dependence over time. We set $B = 10\,000$, $L=25$, and we sample $10\,000$, after a burn-in period of $5\,000$. Visual investigation of the sampled chains supports convergence of the algorithm.

**Fig. 3:**
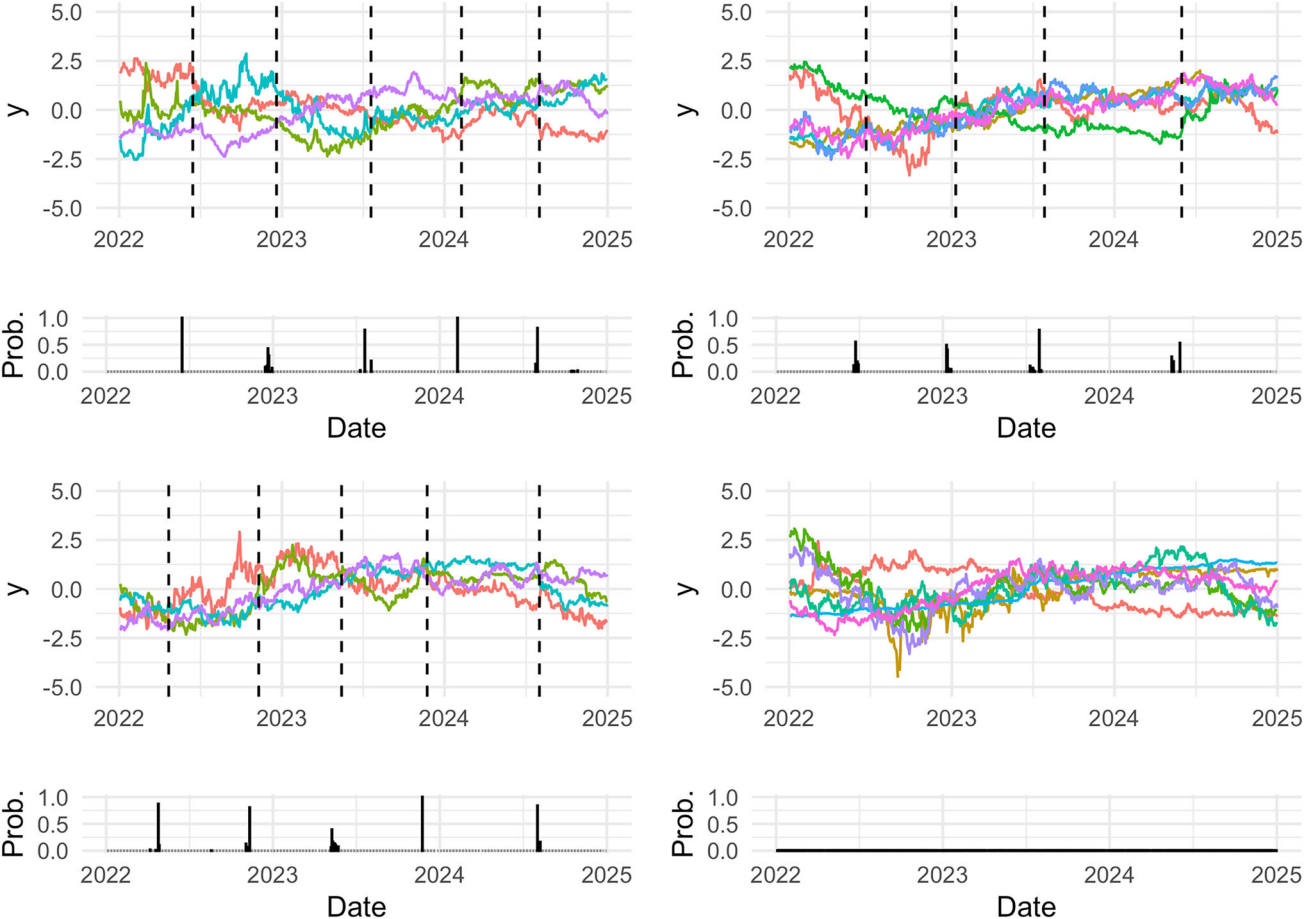
Four largest clusters in the Euro exchange rate data application. In each block, the bottom histogram represents the posterior probability of having a change point at each specific time, while the upper part shows the currencies exchange rates of each cluster, with the cluster-specific marginal change points denoted by the dashed lines

We obtain a point estimate of the latent partition, as the one minimizing the expected Binder loss function, characterized by 9 clusters. Figure 3 shows the four largest clusters with their marginal change points. The largest cluster (bottom-right in Figure 3) includes the Polish Złoty, the Romanian Leu, the Singapore Dollar, the Thai Baht, the Turkish lira, the United States Dollar and the South African Rand. Euro exchange rate with these currencies remained quite stable during the analyzed period, no marginal change points are in fact detected. The second largest cluster (top-right in Figure 3) includes the Hong Kong Dollar, the Japanese Yen, the Mexican Peso, the Norwegian Krone, the New Zealand Dollar and the Philippine peso. They share a change point around June 2022, January 2023, June 2023 and April 2023. One of the other two clusters (top-left in Figure 3) contains the Swiss Franc, the Czech Coruna, the Hungarian Forint and the Israeli Shekel. The other one (bottom-left in Figure 3) includes the British pound sterling, the Icelandic Króna, the Malaysian Ringgit and the Swedish Krona. The marginal change points of these two groups occur at similar times, some of them are very close, others differ only for one or two months. Details about the remaining smaller clusters are shown in Section C of the supplementary material.

## Clustering epidemics with similar structural changes

Next, we apply the previous method to epidemic data. Hereby, we aim to model jointly epidemic spread in different areas, while clustering similar regions on the basis of structural changes in the spreading of the disease. To this end, we consider the standard compartmental susceptible-infected-removed (SIR) model, in which the population is segregated based on immunological statuses of the individuals. In an SIR model, individuals belong to exactly one of the three compartments of susceptible (*S*), infected and infectious (*I*) or recovered/removed (*R*) at any given time point. Under the stochastic law of mass-action (Andersson and Britton 2000), an infected individual infects susceptible individuals whenever an individual Poisson clock rings, before eventually recovering. Once recovered, they play no role in the dynamics of the disease spread.

Let $$\boldsymbol{X}(t) = (X_S(t), X_I(t), X_R(t))$$ where the stochastic processes $$X_S(t), X_I(t)$$, and $$ X_R(t)$$ denote the numbers at time *t* of susceptible, infected and recovered individuals respectively, and take values in the set of non-negative integers $$\mathbb Z_+$$. Since no birth or immigration is assumed into the population, the total population size is conserved at all times, i.e. $$X_S(t)+ X_I(t)+ X_R(t) = X_S(0)+ X_I(0)+ X_R(0)$$ at all times $$t\ge 0$$. Since we are primarily interested in large populations, we use the quantity

12 $$\begin{aligned} \varepsilon = \frac{1}{X_S(0)} \in (0, \infty ) \end{aligned}$$

as a scaling parameter and study the behavior of the system as $\varepsilon \rightarrow 0$. We further assume $\varepsilon X_I(0) \rightarrow I_0 \in (0, 1)$ as $\varepsilon \rightarrow 0$, where the limiting quantity $I_0$ is non-random (deterministic), and assume the initial number of recovered individuals $X_R(0)$ is zero. Let $\beta (t)$ and $\xi (t)$, bounded functions of time *t*, represent the time-varying infection and recovery rates respectively. Let us consider the scaled stochastic process $\boldsymbol{X}^{(\varepsilon )}(t) = (X^{(\varepsilon )}_S(t), X^{(\varepsilon )}_I(t), X^{(\varepsilon )}_R(t))$, where $X^{(\varepsilon )}_S = \varepsilon X_S, X^{(\varepsilon )}_I = \varepsilon X_I$, and $X^{(\varepsilon )}_R = \varepsilon X_R$. The scaled stochastic process $\boldsymbol{X}^{(\varepsilon )}(t)$ is a continuous-time Markov process satisfying

$$\begin{aligned} X^{(\varepsilon )}_S(t)&= X^{(\varepsilon )}_S(0) - M^{(\varepsilon )}_S(t) - \int _0^t \beta (u) X^{(\varepsilon )}_S(u_-) X^{(\varepsilon )}_I(u_-) \textrm{d}u, \\ X^{(\varepsilon )}_I(t)&= X^{(\varepsilon )}_I(0) + M^{(\varepsilon )}_I(t) + \int _0^t \beta (u) X^{(\varepsilon )}_S(u_-) X^{(\varepsilon )}_I(u_-) \textrm{d}u \\&\quad - \int _0^t \xi (u) X^{(\varepsilon )}_I(u_-) \textrm{d}u, \\ X^{(\varepsilon )}_R(t)&= X^{(\varepsilon )}_R(0) + M^{(\varepsilon )}_R(t) + \int _0^t \xi (u) X^{(\varepsilon )}_I(u_-)\textrm{d}u, \end{aligned}$$

where $u_-$ denotes the left-hand limit at *u* and the stochastic processes $M^{(\varepsilon )}_S, M^{(\varepsilon )}_I$, and $M^{(\varepsilon )}_R$ are square-integrable zero-mean martingales, which converge to the zero function in probability as $\varepsilon \rightarrow 0$. See Section B of the supplementary material for a sketch of the derivation of these trajectory equations. Let $||(x_1, x_2, x_3) |\vert _{\infty } = \max \{|{x_1}|, |x_2|, |x_3|\}$. As consequence of the functional law of large numbers, it can be proven that

13 $$\begin{aligned} \lim _{\varepsilon \rightarrow 0} \sup _{t \le T} ||\boldsymbol{X}^{(\varepsilon )}(t) - \bar{\boldsymbol{X}}(t) |\vert _{\infty } {\mathop {\longrightarrow }\limits ^{a.s.}} 0 \end{aligned}$$

where $\bar{\boldsymbol{X}}(t) = (S(t), I(t), R(t))$ is the solution of the following system of ordinary differential equations

14 $$\begin{aligned} \frac{\textrm{d}}{\textrm{d}t}S = - \beta (t) S I, \qquad \frac{\textrm{d}}{\textrm{d}t}I = \beta (t) S I - \xi (t) I , \qquad \frac{\textrm{d}}{\textrm{d}t}R = \xi (t) I, \end{aligned}$$

with the initial condition $$S(0)=1, I(0) = I_0 $$, and $$R(0)=0$$, and some $$T>0$$ (see, e.g., Theorem 3.1 of KhudaBukhsh and Rempała 2024, for a proof).

We note from (13) that the cumulative infection pressure $H_\varepsilon (t)$ converges to a deterministic limit, satisfying

$$\begin{aligned} H_\varepsilon (t) = \int _0^t \beta (u) X^{(\varepsilon )}_I(u) \textrm{d}u {\mathop {\longrightarrow }\limits ^{a.s.}} H(t) = \int _0^t \beta (u) I(u) \textrm{d}u. \end{aligned}$$

Therefore, by virtue of the Sellke construction (e.g., see (Andersson and Britton 2000), and also Section B of the supplementary material), in the limit of the large population the probability that a randomly chosen susceptible individual is still susceptible at time *t* is given by $\exp \left( -H(t)\right)$, which is precisely the function *S*(*t*). That is, the function *S*(*t*) can be interpreted as an improper survival function (improper since $\lim _{t\rightarrow \infty } S(t) >0$) describing the random variable time to infection of a randomly chosen susceptible individual. This forms the basis of the so called dynamical survival analysis (KhudaBukhsh et al. 2023; Rempała and KhudaBukhsh 2023). In practice, we can condition on a final observation time *T* to get a proper survival function, which admits a density

15 $$\begin{aligned} f_{T}(t) = -\left( \frac{1 }{1- S(T)} \right) \frac{\textrm{d}}{\textrm{d}t}S(t) = \frac{\beta (t) S(t) I(t) }{1- S(T)}. \end{aligned}$$

The density in (15) describes the dynamics for a single population in continuous time. A discrete-time analog of the dynamical stochastic analysis method has indeed been developed in Wascher et al. (2024) in the context of monitoring the spread of a disease in a closed population such as a student cohort on campus. Since we are interested in applying our method to data at the level of countries, we decide in favour of a simple model. In practice, this means we will discretize time and use the time-discretized model for clustering purposes at equally-spaced times $t \in \{ 1, \dots , T\}$, which in the following correspond to different days, whereas the infection rate parameter of the generic *i*th observed country $\boldsymbol{\beta }_i = \{\beta _{i,1}, \dots , \beta _{i,T}\}$ changes at certain times described by the specific latent order of the data, while the remaining parameters are assumed to be homogeneous over time. Therefore, given a random sample of daily cases of infections $y_{i,1}, \dots , y_{i,T}$ at a generic *i*th location, observed up to some terminal time $T>0$, the likelihood contribution of the *i*th observed series is given by

16 $$\begin{aligned} \mathcal {L}(\boldsymbol{y}_i \mid \boldsymbol{\beta }_i, \rho _i) = \prod _{j=1}^{m_i}\prod _{t=t_{i,j}^-}^{t_{i,j}^+} f_{i,T}(t)^{y_{i,t}},\quad i=1, \dots , n, \end{aligned}$$

where $f_{i,T}(t)$ is defined in (15) and obtained by integrating numerically the system in (14) with infection rate $\boldsymbol{\beta }_i$. This is the likelihood function we will use for our clustering inference.

We remark that, also for the epidemiological case, we are not interested in inferring parameter values but on the latent clustering structure of the data. However, while in Section 5 of the supplementary material we could integrate analytically the parameters of the kernel function, here we resort to numerical integration of those parameters. Specifically, we assume the recovery rate $\xi$ to be constant and shared across different infection time series. The parameter $I_0$ can be updated through an observation-specific Metropolis–Hastings step. Finally, the infection rates $\boldsymbol{\beta }_i$s, which change over time, are marginalized via Monte Carlo integration.

### Synthetic infection study

**Fig. 4:**
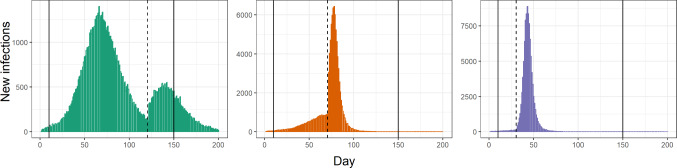
An example of bar plots of daily new cases in the synthetic study. Different panels correspond to different populations, one for each cluster considered in the study

We first show through a simulation study the performance of the proposed model in an epidemiological scenario. Data are generated using the Doob–Gillespie algorithm (see Anderson and Kurtz 2015, and Section C of the supplementary material for pseudo-code). We consider a set of $n = 10$ populations, where the starting number of susceptibles is $S_0 = 100\,000$. We keep the recovery rate fixed at $\xi = 1/8$, we consider a time-varying rate of infection $\beta _{i,t}$ and a different starting proportion of infected $I_{i,0}$ for each population. The simulated data are generated in a time period of 200 days, however in the analysis we consider a restricted window of time to remove the tails of the epidemic. We restrict the window from time $t = 10$ to time $t = 150$ thus resulting in a time period of length $T = 140$. We assume three different groups where the parameters are as shown in Table 1 of Section C of the supplementary material. In the first group we simulate an epidemic where we have two large spreads, while in the second and third group we simulate a single large spread but with different change points. Figure 4 shows an example of simulated data for each of the groups considered in the study.

We investigate how different parameters affect posterior estimates and the algorithm performance. Specifically, we vary the number of points used to marginalize via Monte Carlo the observation-specific infection rates, here denoted by MC, as well as the accuracy of the normalization constant $\mathrm B$ and depth of the proposal $\mathrm L$. We set $\xi = 1/8$ to be consistent with the parameters we used to generate synthetic data. Further, we marginalize the infection rate whereas the Monte Carlo integration is performed by sampling the unique values in $\boldsymbol{\beta }_i$ from a gamma distribution with (4, 10) as shape and rate parameters. Finally, for each population we randomly sample without replacement 20% of the infection times, since the dynamical survival analysis method requires only a subset of infections (see KhudaBukhsh et al. 2023). In each replicate, we run the algorithm for $5\,000$ iterations, discarding $2\,000$ as burn-in period. The point estimate of the latent partition $\hat{\lambda }$ is then obtained as minimizer of the expected Binder loss function (Binder 1978) among those visited by the algorithm. To assess the algorithm performance and the quality of the posterior estimates, we use the Binder loss between the posterior point estimates and the true latent partitions of the data, reported in Table 3.

**Table 3:** Posterior summaries of the synthetic epidemiological data. Different scenarios are obtained combining different accuracies of the numerical integration of the infection rates $\textrm{MC}$, different accuracies of the normalization constant $\mathrm B$ and different proposal depths $\mathrm L$. The table reports the Binder loss between the point estimate and the true latent partition of the data (BI). Results are averaged over 50 replicates

$\textrm{MC} = 250$			$\textrm{MC} = 500$			$\textrm{MC} = 1\,000$		
$\mathrm B$	$\mathrm L$	$\textrm{BI}(\hat{\lambda }, \lambda _0)$	$\mathrm B$	$\mathrm L$	$\textrm{BI}(\hat{\lambda }, \lambda _0)$	$\mathrm B$	$\mathrm L$	$\textrm{BI}(\hat{\lambda }, \lambda _0)$
$10^3$	1	0.345	$10^3$	1	0.200	$10^3$	1	0.005
	25	0.294		25	0.144		25	0.010
	100	0.257		100	0.194		100	0.018
$10^4$	1	0.354	$10^4$	1	0.234	$10^4$	1	$\ll 10^{-4}$
	25	0.323		25	0.042		25	0.010
	100	0.281		100	0.162		100	0.008
$10^5$	1	0.320	$10^5$	1	0.280	$10^5$	1	0.014
	25	0.310		25	0.249		25	0.007
	100	0.212		100	0.092		100	0.009

We can appreciate a similar behaviour to the one observed in the time series synthetic study: also with a complex kernel function, such as an SIR model, posterior estimates and performance of the algorithm are not particularly affected by the normalization constant accuracy $\mathrm B$ and the proposal depth $\mathrm L$. However, increasing the number of points used for the Monte Carlo marginalization of the infection rate (MC) has a positive effect on the quality of the partition estimates. In particular, for $\mathrm MC = 1000$ we obtain posterior estimates having comparable precision to the time series synthetic study (Section 5), where local parameters are marginalized analytically.

### SARS-CoV-2 Europe infection data

We now consider the real data analysis motivating the model we study in this manuscript. Our aim is to identify possible clusters of homogeneous states among the 27 countries that comprise the European Union, with respect to the spread of SARS-CoV-2 virus. The first confirmed case was in Bordeaux (France) on the 24th January 2020, while the first major outbreak was experienced in Italy in late February, and different countries within the European Union experienced different starting time of the spreading. Although the response to the pandemic was different from one country to another, some actions were taken jointly with the European commission such as travel ban and vaccination.

Hereby, we consider infection data taken from the European Centre for Disease Prevention and Controls (2022) database on COVID-19 daily new cases, for a time period of more than one year that goes from 1st June 2021 to 30th September 2022. We selected this specific time window to mitigate differences of the pandemic, as it starts after the beginning of the world mass immunization campaigns and includes the largest spread of new detected case in Europe that lasted from the end of 2021 to September 2022. We smooth the data by computing for each day the 7-days rolling average of the number of infected. By doing so, we remove some common measurement errors that occur when counting the number of daily new cases, e.g. the number of weekend recorded daily new cases is always smaller than during week days. We set $\xi = 1/6$ and the unique values of the infection rates out of each $\boldsymbol{\beta }_i$ are sampled from a gamma distribution with (2, 10) as shape and rate parameters. We increase the accuracy of the normalization constants to $B = 100\,000$, since we have $T = 487$ days, we set $L=1$ to reduce the computational time, and $MC = 1\,000$. Finally, we do not consider the entire data, but a subset of $50\,000$ infection times randomly sampled for each country. The MCMC algorithm is run for $12\,500$ iterations of which $2\,500$ have been discarded as burn-in period.

**Fig. 5:**
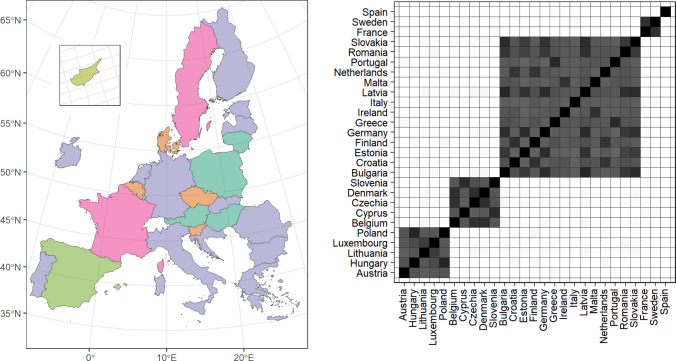
Summaries of the EU27 countries analysis. Left plot: countries colored consistently with the point estimate of the latent partition. Right plot: posterior similarity matrix

The posterior point estimate of the latent partition of the data is composed of five clusters, which are shown in Figure 5, along with the posterior similarity matrix. We have a large cluster with 14 countries, two clusters with 5 countries, one with two countries and one with just one country. The posterior similarity matrix in Figure 5 shows that the clusters are well separated, moreover, a visual check confirms convergence of the posterior chain. By analyzing the change point patterns of each cluster - available through the empirical survival functions in Section A of the supplementary material - we can infer the common features shared by countries grouped together. We identify four time periods in which most of the change points occur. The first is summer 2021 where the number of infections slightly increased after the curve was flattened by mass vaccination, here all clusters exhibit at least one change point in this phase. The second period occurs in winter 2021-2022, when the Omicron variant started spreading. Clusters 1 and 2 have both a change point in January 2022, while Cluster 3 - the largest one - and Cluster 4 - which consists only of Spain - have an early change point in September when the number of infections gradually started to increase, reaching a peak at the beginning of 2022. In the same period France and Sweden experience two distinct change points, one in December and one in February. Later, in spring 2022, we can identify another set of change points; in particular, Clusters 3 and 4 have a change point in April and Cluster 3 in May. Finally in summer 2022 we detect change points for Spain, a highly popular tourist destination, as well as for Cluster 3 (which includes Italy and Greece) and Cluster 2.

## Discussion

In the previous sections, motivated by an epidemiological study, we introduced a novel approach to cluster together time-dependent observations having the same structural change times. Such approach works on a mixture representation of the model, whereas the mixing parameters is a random order of the observational times, which induce locally homogeneous behaviors but also change points. The synthetic studies we presented have shown the performance of our proposal with respect to other competitor, favoring the first when performing model-based clustering on change points. Beyond the cases we considered in the manuscript, this modeling approach can be easily adapted to several frameworks where the interest lies in defining groups of time-dependent observations.

Along the manuscript, we have assumed a symmetric prior distribution. However, this assumption can be relaxed by considering prior information associated with specific latent orders, in the spirit of Canale et al. (2017). Further, instead of a Dirichlet-multinomial model, one can consider approaches based on more flexible finite-dimensional discrete prior distributions, in the spirit of Lijoi et al. (2020, 2024). However, the computational complexity of these approaches may result in models for time series clustering that are challenging to handle.

Further, there is no borrowing of information across different cluster. While this assumption improves the model’s tractability, having possibly dependent orders increase its flexibility. For example, recent contributions in this direction can be found in Quinlan et al. (2024). In this manuscript we considered an approach based on the inspiring studies of Martínez and Mena (2014), where the authors restrict an EPPF from the partitions’ space to the orders’ space. In the same spirit, one can consider restricting a partially exchangeable partition probability function (see, e.g., Pitman 1995) from the partitions’ space to the orders’ space, obtaining a distribution that introduces dependence among different atoms. The model discussed in this paper can then be extended by considering such a distribution to introduce dependencies among different dimensions, but preserving the same combinatorial structure and flexibility.

## Supplementary Information

Below is the link to the electronic supplementary material.

Supplementary file 1 (pdf 577 KB)

## Data Availability

All data are open and freely accessible online.

## Footnotes

1 Data are available in the European Central Bank website at https://www.ecb.europa.eu/stats/policy_and_exchange_rates/euro_reference_exchange_rates/html/index.en.html.
