# Supplementary material for: Model-based clustering of time-dependent observations with common structural changes
Source: Stat Comput. 2025 Oct 28;36(1):7. doi: 10.1007/s11222-025-10756-x (PMC12568813; doi:10.1007/s11222-025-10756-x)
Supplement: Supplementary file 1 — (pdf 577 KB) [file 11222_2025_10756_MOESM1_ESM.pdf]

Supplementary material for "Model-based clustering of time-dependent observations with common structural changes"

Corradin R.*[1], Danese L.†[1], KhudaBukhsh W. R. ‡[2], and Ongaro A.§[1]

[1]Department of Economics, Management and Statistics, University of Milano-Bicocca, Milano 20136, Italy
[2]School of Mathematical Sciences, University of Nottingham, Nottingham NG7 2RD, U.K.

A Additional details on the split-merge algorithm

In this section we provide further details on Algorithm 1, which can be helpful for future implementations of the model discussed in the manuscript. The split-merge strategy we used in the manuscript is based on the preliminary studies of Jain and Neal [2004, 2007], where we make use of some results of the latter for non-conjugate distributions. Let us first recall the general specification of the model, with

$$\begin{aligned} \boldsymbol{y}_i \mid \rho_i, \boldsymbol{\theta}_i^* &\sim \prod_{j=1}^{m_i} \prod_{t=t_{i,j}^-}^{t_{i,j}^+} \mathcal{L}(y_{i,t} \mid y_{i,t-1}, \theta_{i,j}^*), \quad i = 1, \ldots, n, \\ \rho_i \mid \tilde{p}(\rho) &\overset{\text{iid}}{\sim} \tilde{p}(\rho), \quad i = 1, \ldots, n, \\ (\pi_1, \ldots, \pi_{2^{T-1}}) &\sim \textsc{Dir}(\alpha_1, \ldots, \alpha_{2^{T-1}}), \\ \theta_{i,j}^* &\overset{\text{iid}}{\sim} P_0(\theta), \quad j = 1, \ldots, m_i, \; i = 1, \ldots, n, \end{aligned} \tag{S1}$$

whereas

$$\tilde{p}(\rho) = \sum_{r=1}^{2^{T-1}} \pi_r \delta_{\tilde{\rho}_r}(\rho), \tag{S2}$$

$\{\tilde{\rho}_1, \ldots, \tilde{\rho}_{2^{T-1}}\}$ denote all the possible orders of T elements, and $(\pi_1, \ldots, \pi_{2^{T-1}})$ is a vector of probabilities taking values in the $(2^{T-1} - 1)$-dimensional simplex space.

Before discussing the sampling step, we introduce some quantities which will be needed in the derivation of the acceptance rates. We first note that the distribution of a sequence of elements $\mathcal{R} = \{\rho_1, \ldots, \rho_n\}$ can be decomposed into the distribution of the partition structure λ times the distribution of the unique elements $\mathcal{R}^* = \{\rho_1^*, \ldots, \rho_k^*\}$, with $k \le n$, and

$$\mathcal{L}(\mathcal{R}) = \mathcal{L}(\lambda)\mathcal{L}(\mathcal{R}^*).$$

The first term is given by the EPPF of a Dirichlet-categorical model, with

$$\mathcal{L}(\lambda) = \frac{\Gamma(\alpha^+)}{\Gamma(\alpha^+ + n)} \prod_{r=1}^{k} \frac{\Gamma(\alpha_r + n_r)}{\Gamma(\alpha_r)},$$

with $\lambda = \{B_1, \ldots, B_k\}$ and $n_j = |B_j|$. The second term is the probability associated with a specific set of unique values. As far as all the values have the same prior guess, the joint distribution of a specific subset is given by the law of a uniform sampling without replacement. Hence, we have

$$\mathcal{L}(\mathcal{R}^*) = 1 - \prod_{j=0}^{k-1} \left(1 - \frac{1}{2^{T-1} - j}\right),$$

*riccardo.corradin@unimib.it
†luca.danese1@campus.unimib.it
‡wasiur.khudabukhsh@nottingham.ac.uk
§andrea.ongaro@unimib.it

where k is the number of groups. Finally, we denote by

$$q(\lambda^{(N)}, \mathcal{R}^{(N)} \mid \lambda^{(O)}, \mathcal{R}^{(O)}, \mathcal{Y})$$

the proposal from a previous state $(\lambda^{(O)}, \mathcal{R}^{(O)})$ to a new state $(\lambda^{(N)}, \mathcal{R}^{(N)})$, where we emphasize the dependence on $\mathcal{Y}$ of the mixture strategy as proposal distribution for the new orders. Further, we have that the proposal factorises into two terms

$$q(\lambda^{(N)}, \mathcal{R}^{(N)} \mid \lambda^{(O)}, \mathcal{R}^{(O)}, \mathcal{Y}) = q(\lambda^{(N)} \mid \lambda^{(O)}) q(\mathcal{R}^{(N)} \mid \mathcal{Y}), \tag{S3}$$

whereas the first term in the left side of the previous equation is described in Jain and Neal [2004] and does not depend on $\mathcal{Y}$, while the second term is a product of independent proposals following the mixture of posteriors discussed in the manuscript, i.e.

$$\psi(\rho \mid \mathcal{Y}) = \sum_{i=1}^{n} \frac{1}{n} \mathcal{L}(\rho \mid \boldsymbol{y}_i). \tag{S4}$$

We can now describe a detailed single step of the split-merge strategy for clustering multiple time series. Suppose we are performing the generic m step of the algorithm to cluster the observed data $\mathcal{Y}$, assuming we are on a current state of the chain, with $\lambda^{(m-1)} = \{B_1^{(m-1)}, \dots, B_k^{(m-1)}\}$ being the latent partition of the data in k groups at the current state and $\mathcal{R}^{*(m-1)} = \{\rho_1^{*(m-1)}, \dots, \rho_k^{*(m-1)}\}$ denotes the unique values of the latent orders out of $\mathcal{R}^{(m-1)} = \{\rho_1^{(m-1)}, \dots, \rho_n^{(m-1)}\}$, associated with each cluster in $\lambda^{(m-1)}$.

We initialize the proposed values $\lambda^{(N)}$ and $\mathcal{R}^{(N)}$ by setting the latent partition and the unique values of the random orders equal to the previous state values, i.e. we set $\lambda^{(N)} = \lambda^{(m-1)}$ and $\mathcal{R}^{*(N)} = \mathcal{R}^{*(m-1)}$. The split-merge step starts by sampling two indices $i \neq \ell$ from $\{1, \dots, n\}$. Then, one of the following cases can occur.

(a) If the observations i and ℓ belong to the same cluster, e.g. to the generic s block $B_s^{(m-1)}$ of $\lambda^{(m-1)}$, then we perform a split step.

- We update the proposed partition $\lambda^{(N)}$ according to the split step: we assign i to $B_s^{(N)}$ and ℓ to a new block $B_{k+1}^{(N)}$. We then randomly assign all the remaining observations in $B_s^{(m-1)} \setminus \{i, \ell\}$ to $B_S^{(N)}$ or $B_{k+1}^{(N)}$.
- We update the proposed unique values of the random orders $\mathcal{R}^{*(N)}$, whereas we first increase the dimension of $\mathcal{R}^{*(N)}$ to $k+1$ and then the elements s and $k+1$ are sampled from the mixture of posterior distributions in (S4).
- Compute the acceptance rate

$$\alpha(\lambda^{(N)}, \mathcal{R}^{*(N)} \mid \lambda^{(m-1)}, \mathcal{R}^{*(m-1)}) = \min\left\{1, \frac{q(\lambda^{(m-1)}, \mathcal{R}^{*(m-1)} \mid \lambda^{(N)}, \mathcal{R}^{*(N)})}{q(\lambda^{(N)}, \mathcal{R}^{*(N)} \mid \lambda^{(m-1)}, \mathcal{R}^{*(m-1)})} \right.$$
$$\left. \times \frac{\mathcal{L}(\lambda^{(N)}) \mathcal{L}(\mathcal{R}^{*(N)})}{\mathcal{L}(\lambda^{(m-1)}) \mathcal{L}(\mathcal{R}^{*(m-1)})} \frac{\mathcal{L}(\mathcal{Y} \mid \lambda^{(N)}, \mathcal{R}^{*(N)})}{L(\mathcal{Y} \mid \lambda^{(m-1)}, \mathcal{R}^{*(m-1)})} \right\}.$$

Thanks to the factorization in (S3), the first ratio in the acceptance rate becomes

$$\frac{q(\lambda^{(m-1)} \mid \lambda^{(N)})}{q(\lambda^{(N)} \mid \lambda^{(m-1)})} \frac{q(\mathcal{R}^{(m-1)} \mid \mathcal{Y})}{q(\mathcal{R}^{(N)} \mid \mathcal{Y})} = 2^{|B_s^{(m-1)}|-2} \frac{\psi(\rho_s^{*(m-1)} \mid \mathcal{Y})}{\psi(\rho_s^{*(N)} \mid \mathcal{Y}) \psi(\rho_{k+1}^{*(N)} \mid \mathcal{Y})}$$

where the first term follows the derivation in Jain and Neal [2004] while the second is given by the mixture of posteriors proposal distributions. For the remaining terms, the followings hold

$$\frac{\mathcal{L}(\lambda^{(N)})}{\mathcal{L}(\lambda^{(m-1)})} = \frac{\Gamma\big(\alpha + n_s^{(N)}\big) \Gamma\big(\alpha + n_{k+1}^{(N)}\big)}{\Gamma(\alpha) \Gamma\big(\alpha + n_s^{(m-1)}\big)}$$

$$\frac{\mathcal{L}(\mathcal{R}^{*(N)})}{\mathcal{L}(\mathcal{R}^{*(m-1)})} = \frac{1 - \prod_{j=0}^{k} \left(1 - \frac{1}{2^{T-1} - j}\right)}{1 - \prod_{j=0}^{k-1} \left(1 - \frac{1}{2^{T-1} - j}\right)}$$

$$\frac{\mathcal{L}(\mathcal{Y} \mid \lambda^{(N)}, \mathcal{R}^{*(N)})}{\mathcal{L}(\mathcal{Y} \mid \lambda^{(m-1)}, \mathcal{R}^{*(m-1)})} = \frac{\prod_{j \in B_s^{(N)}} \mathcal{L}\big(\boldsymbol{y}_j \mid \rho_s^{*(N)}\big) \prod_{j \in B_{k+1}^{(N)}} \mathcal{L}\big(\boldsymbol{y}_j \mid \rho_{k+1}^{*(N)}\big)}{\prod_{j \in B_s^{(m-1)}} \mathcal{L}\big(\boldsymbol{y}_j \mid \rho_s^{*(m-1)}\big)}$$

where $n_s^{(N)}$ and $n_s^{(m-1)}$ denote the size of the sth block in the proposed partition and the previous state, respectively, and the latter equation follows the restricted Gibbs sampling strategy discussed in Jain and Neal [2004].

(b) If observations i and ℓ belong to different clusters, e.g. $i \in B_s^{(m-1)}$ and $\ell \in B_r^{(m-1)}$ with $s < r$, then we perform a merge step.

- We update the proposed partition $\lambda^{(N)}$ according to the merge step: we assign all the observations in $B_r^{(m-1)}$ to $B_s^{(m-1)}$ and we destroy the block $B_r^{(m-1)}$.
- We update the proposed state for the unique values of the random orders $\mathcal{R}^{*(N)}$ by destroying the rth element and by sampling the sth element from the mixture of posterior distributions in (S4).
- Compute the acceptance rate

$$\alpha(\lambda^{(N)}, \mathcal{R}^{*(N)} \mid \lambda^{(m-1)}, \mathcal{R}^{*(m-1)}) = \min\left\{1, \frac{q(\lambda^{(m-1)}, \mathcal{R}^{*(m-1)} \mid \lambda^{(N)}, \mathcal{R}^{*(N)})}{q(\lambda^{(N)}, \mathcal{R}^{*(N)} \mid \lambda^{(m-1)}, \mathcal{R}^{*(m-1)})} \right.$$
$$\left. \times \frac{\mathcal{L}(\lambda^{(N)})\mathcal{L}(\mathcal{R}^{*(N)})}{\mathcal{L}(\lambda^{(m-1)})\mathcal{L}(\mathcal{R}^{*(m-1)})} \frac{\mathcal{L}(\mathcal{Y} \mid \lambda^{(N)}, \mathcal{R}^{*(N)})}{L(\mathcal{Y} \mid \lambda^{(m-1)}, \mathcal{R}^{*(m-1)})} \right\}.$$

Similarly to point (a), we have

$$\frac{q(\lambda^{(m-1)} \mid \lambda^{(N)})}{q(\lambda^{(N)} \mid \lambda^{(m-1)})} \frac{q(\mathcal{R}^{(m-1)} \mid \mathcal{Y})}{q(\mathcal{R}^{(N)} \mid \mathcal{Y})} = \left(\frac{1}{2}\right)^{|B_s^{(m-1)}|+|B_r^{(m-1)}|-2} \frac{\psi(\rho_s^{*(m-1)} \mid \mathcal{Y})\psi(\rho_r^{*(m-1)} \mid \mathcal{Y})}{\psi(\rho_s^{*(N)} \mid \mathcal{Y})}.$$

Further, for the remaining terms the followings hold

$$\frac{\mathcal{L}(\lambda^{(N)})}{\mathcal{L}(\lambda^{(m-1)})} = \frac{\Gamma(\alpha)\Gamma\big(\alpha + n_s^{(N)}\big)}{\Gamma\big(\alpha + n_s^{(m-1)}\big)\Gamma\big(\alpha + n_r^{()m-1}\big)},$$
$$\frac{\mathcal{L}(\mathcal{R}^{*(N)})}{\mathcal{L}(\mathcal{R}^{*(m-1)})} = \frac{1 - \prod_{j=0}^{k-1}\left(1 - \frac{1}{2^{T-1}-j}\right)}{1 - \prod_{j=0}^{k}\left(1 - \frac{1}{2^{T-1}-j}\right)},$$
$$\frac{\mathcal{L}(\mathcal{Y} \mid \lambda^{(N)}, \mathcal{R}^{*(N)})}{\mathcal{L}(\mathcal{Y} \mid \lambda^{(m-1)}, \mathcal{R}^{*(m-1)})} = \frac{\prod_{j \in B_s^{(N)}} \mathcal{L}\big(\boldsymbol{y}_j \mid \rho_s^{*(N)}\big)}{\prod_{j \in B_s^{(m-1)}} \mathcal{L}\big(\boldsymbol{y}_j \mid \rho_s^{*(m-1)}\big) \prod_{j \in B_r^{(m-1)}} \mathcal{L}\big(\boldsymbol{y}_j \mid \rho_{k+1}^{*(m-1)}\big)}.$$

Finally, we perform a Metropolis–Hasting step by sampling a uniform random variable $U \sim Unif(0,1)$. If $U < \alpha(\lambda^{(N)}, \mathcal{R}^{*(N)} \mid \lambda^{(m-1)}, \mathcal{R}^{*(m-1)})$ we accept the proposed values $\lambda^{(N)}$ and $\mathcal{R}^{*(N)}$ as current state of the chain, setting $(\lambda^{(m)}, \mathcal{R}^{*(m)})$ equal to $(\lambda^{(N)}, \mathcal{R}^{*(N)})$, otherwise if $U > \alpha(\lambda^{(N)}, \mathcal{R}^{*(N)} \mid \lambda^{(m-1)}, \mathcal{R}^{*(m-1)})$ we stay on the previous state of the chain and we set $(\lambda^{(m)}, \mathcal{R}^{*(m)})$ equal to $(\lambda^{(m-1)}, \mathcal{R}^{*(m-1)})$.

B Additional details on the SIR model

The scaled stochastic process $\boldsymbol{X}^{(\varepsilon)}$ is a time-inhomogeneous continuous-time Markov process generated by Gikhman and Skorokhod [2004, Chapter 1] (see also Applebaum [2019], Engel and Nagel [2000])

$$A_t^{(\varepsilon)} g(x) = \varepsilon^{-1}\beta(t)x_1x_2(g(x_1 - \varepsilon, x_2 + \varepsilon, x_3) - g(x)) + \varepsilon^{-1}\xi(t)x_2(g(x_1, x_2 - \varepsilon, x_3 + \varepsilon) - g(x)), \tag{S5}$$

for bounded, continuous functions $g : \mathbb{R}_+^3 \mapsto \mathbb{R}$. The trajectories of the scaled process $X^{(\varepsilon)}$ can be described as solutions to the following stochastic differential equations (written in the integral form)

$$\begin{aligned}
X_S^{(\varepsilon)}(t) &= X_S^{(\varepsilon)}(0) - \varepsilon \int_0^t \int_0^\infty \mathbb{1}_{[0, \varepsilon^{-1}\beta(u)X_S^{(\varepsilon)}(u_-)X_I^{(\varepsilon)}(u_-)]}(v)\, Q_1(\mathrm{d}u, \mathrm{d}v), \\
X_I^{(\varepsilon)}(t) &= X_I^{(\varepsilon)}(0) + \varepsilon \int_0^t \int_0^\infty \mathbb{1}_{[0, \varepsilon^{-1}\beta(u)X_S^{(\varepsilon)}(u_-)X_I^{(\varepsilon)}(u_-)]}(v)\, Q_1(\mathrm{d}u, \mathrm{d}v) \\
&\quad - \varepsilon \int_0^t \int_0^\infty \mathbb{1}_{[0, \varepsilon^{-1}\xi(u)X_I^{(\varepsilon)}(u_-)]}(v)\, Q_2(\mathrm{d}u, \mathrm{d}v), \\
X_R^{(\varepsilon)}(t) &= X_R^{(\varepsilon)}(0) + \varepsilon \int_0^t \int_0^\infty \mathbb{1}_{[0, \varepsilon^{-1}\xi(u)X_I^{(\varepsilon)}(u_-)]}(v)\, Q_2(\mathrm{d}u, \mathrm{d}v),
\end{aligned} \tag{S6}$$

where Q_1, Q_2 are independent Poisson random measures on $\mathbb{R}^2$ with intensity measure $\mathrm{d}u \times \mathrm{d}v$ where $\mathrm{d}u$, and $\mathrm{d}v$ are Lebesgue measures on $\mathbb{R}$. We have used the notation $\mathbb{1}_A(x)$ to denote the indicator function of a set A, which takes the value one when $x \in A$, and zero otherwise. The process X (or the scaled process $X^{(\varepsilon)}$) can simulated as a pure jump process using standard techniques. An alternative individual-based approach is provided by what is known as the *Sellke construction* Andersson and Britton [2000]. Let $H_\varepsilon(t)$ denote the cumulative infection pressure upto time t, where

$$H_\varepsilon(t) = \int_0^t \varepsilon\beta(u)X_I(u)\mathrm{d}u = \int_0^t \beta(u)X_I^{(\varepsilon)}(u)\mathrm{d}u. \tag{S7}$$

According to the Sellke construction, each susceptible individual is assigned an exponentially distributed (with mean one) threshold E, and gets infected at time t as soon as $E \le H_\varepsilon(t)$. If such a time never arrives, *i.e.*, if $E > \lim_{t\to\infty} H_\varepsilon(t)$ (the limit is finite almost surely), then the individual escapes infection. That is, conditional on the history of the process up to time t, the probability that a randomly chosen susceptible individual is still susceptible at time t, *i.e.*, has not been infected by time t, is $\exp(-H_\varepsilon(t))$. This individual-based perspective is at the heart of the Dynamical Survival Analysis (DSA) KhudaBukhsh et al. [2020], Di Lauro et al. [2022] approach to parameter inference for infectious disease epidemiology based on a random sample of infection times (and recovery times, if available) as opposed to standard methods that require (or impute when full data are not available) population-level trajectories.

To study the large population limit of the scaled stochastic process $X^{(\varepsilon)}$, let us rewrite the trajectory in (S6) as

$$\begin{aligned}
X_S^{(\varepsilon)}(t) &= X_S^{(\varepsilon)}(0) - M_S^{(\varepsilon)}(t) - \int_0^t \beta(u)X_S^{(\varepsilon)}(u_-)X_I^{(\varepsilon)}(u_-)\mathrm{d}u,\\
X_I^{(\varepsilon)}(t) &= X_I^{(\varepsilon)}(0) + M_I^{(\varepsilon)}(t) + \int_0^t \beta(u)X_S^{(\varepsilon)}(u_-)X_I^{(\varepsilon)}(u_-)\mathrm{d}u - \int_0^t \xi(u)X_I^{(\varepsilon)}(u_-)\mathrm{d}u,\\
X_R^{(\varepsilon)}(t) &= X_R^{(\varepsilon)}(0) + M_R^{(\varepsilon)}(t) + \int_0^t \xi(u)X_I^{(\varepsilon)}(u_-)\mathrm{d}u,
\end{aligned}$$

where the stochastic processes $M_S^{(\varepsilon)}, M_I^{(\varepsilon)}$, and $M_R^{(\varepsilon)}$ are square-integrable zero-mean martingales defined as

$$\begin{aligned}
M_S^{(\varepsilon)}(t) &= \varepsilon \int_0^t \int_0^\infty \mathbb{1}_{[0,\varepsilon^{-1}\beta(u)X_S^{(\varepsilon)}(u-)X_I^{(\varepsilon)}(u-)]}(v)\,\bar{Q}_1(\mathrm{d}u,\mathrm{d}v),\\
M_I^{(\varepsilon)}(t) &= \varepsilon \int_0^t \int_0^\infty \mathbb{1}_{[0,\varepsilon^{-1}\beta(u)X_S^{(\varepsilon)}(u-)X_I^{(\varepsilon)}(u-)]}(v)\,\bar{Q}_1(\mathrm{d}u,\mathrm{d}v) - \varepsilon \int_0^t \int_0^\infty \mathbb{1}_{[0,\varepsilon^{-1}\xi(u)X_I^{(\varepsilon)}(u-)]}(v)\,\bar{Q}_2(\mathrm{d}u,\mathrm{d}v),\\
M_R^{(\varepsilon)}(t) &= \varepsilon \int_0^t \int_0^\infty \mathbb{1}_{[0,\varepsilon^{-1}\xi(u)X_I^{(\varepsilon)}(u-)]}(v)\,\bar{Q}_2(\mathrm{d}u,\mathrm{d}v),
\end{aligned}$$

where $\bar{Q}_1$ and $\bar{Q}_2$ are the compensated Poisson random measures (corresponding to Q_1 and Q_2 respectively). It is easy to verify that the quadratic variations of the above martingales all converge to zero in probability as $\varepsilon \to 0$, which in turn imply that the martingales themselves converge to the deterministic function taking the value zero at all times. Consider the system of Ordinary Differential Equations (ODEs) in (14) with the initial condition $S(0) = 1, I(0) = I_0$, and $R(0) = 0$. Let $\bar{X} = (S, I, R)$. Then, note that $X^{(\varepsilon)}(0) \to (1, I_0, 0)$ as $\varepsilon \to 0$ by design, and as a consequence of the Functional Law of Large Numbers (FLLN), we can prove that

$$\lim_{\varepsilon\to 0} \sup_{t\le T} ||X^{(\varepsilon)}(t) - \bar{X}(t)||_\infty \to 0 \tag{S8}$$

in probability where $||(x_1, x_2, x_3)||_\infty = \max\{|x_1|, |x_2|, |x_3|\}$. In fact, the above convergence holds almost surely since we have assumed the functions $\beta(t)$ and $\xi(t)$ are bounded. The proof follows by standard arguments using Grönwall's inequality and some maximal estimates. See, for example, KhudaBukhsh and Rempała [2024, Theorem 3.1], where it is also discussed how an SIR model with time-varying infection and recovery rates such as the model we use in this paper can be used to approximate a compartmental Susceptible-Exposed-Infected-Recovered (SEIR) model.

C Additional figures, tables and algorithms

In this section we report additional materials to support the manuscript, such as figures and algorithms. First, we show in Figure 1 an example of simulated data for the time series synthetic study. The data generating

process and observation-specific parameters are described in Section 5. The figure shows in the left panel different time series, whereas each series is an observation characterized by its local parameters, but some of the series share the same structural change times. The right part of the figure shows two posterior similarity matrices, associated with two distinct estimates of the model, with the same accuracy of the normalization constant $B = 10\,000$, but varying the depth of the proposal, top with $L = 1$ and bottom with $L = 100$.

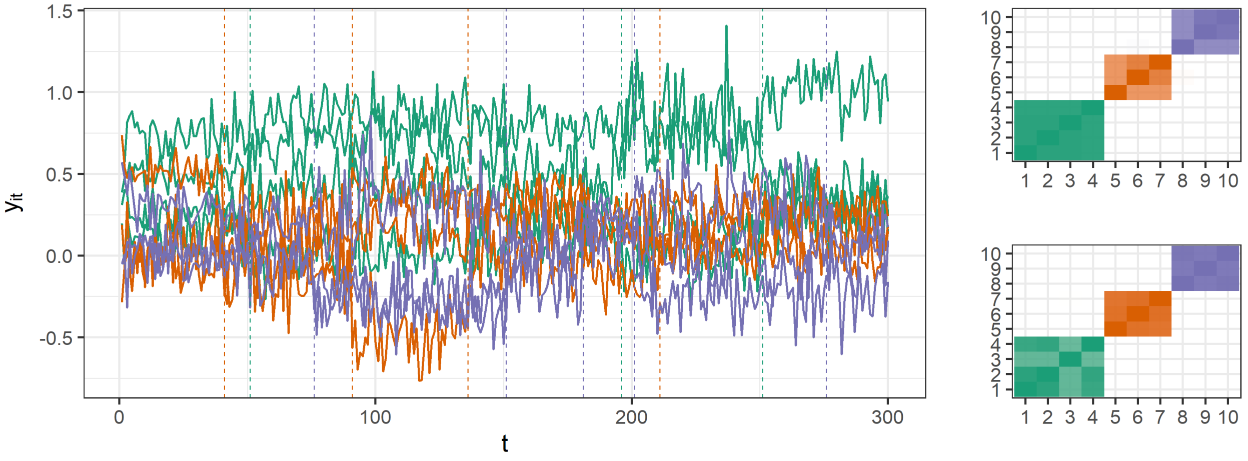

Figure 1: Left panel: one realization of simulated data according to the model in (9) and parameters in Table 1. Right panels: posterior similarity matrix of one replication with $B = 10\,000$, top with $L = 1$ and bottom with $L = 100$ respectively.

Regarding the application to exchange rate data in Section 5.1, Figure 2 reports the marginal change points of the five smallest estimated clusters.

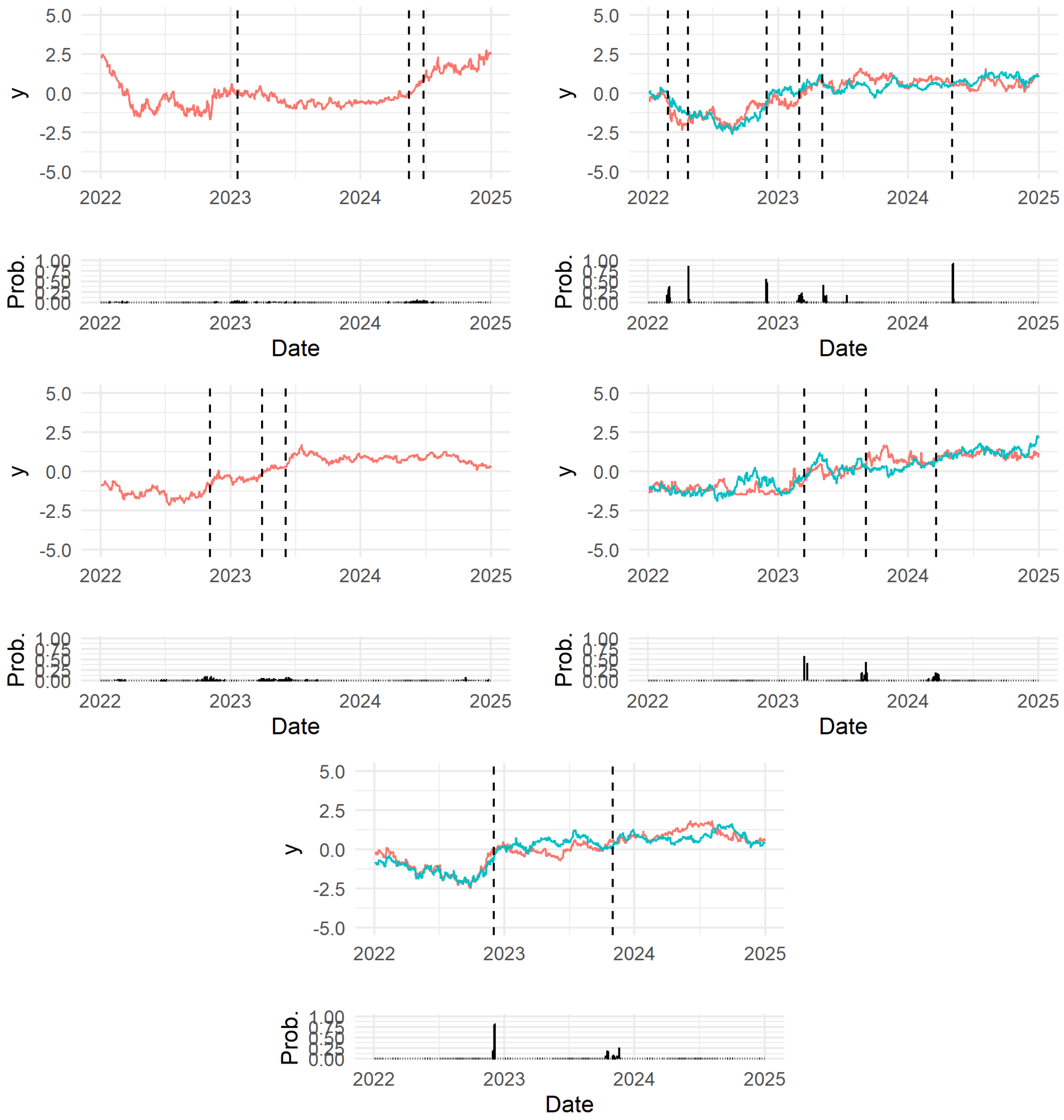

Figure 2: Marginal change points for the five smallest clusters of the application on Euro exchange rates. In each block the histogram in the lower part represents the frequency of times that each time point has been detected as a change point in the MCMC chain, in the upper block are represented the exchange rates of each group with the marginal change points of the cluster denoted by the dashed lines.

For the application to COVID-19 real data in Section 6.2, Figure 3 shows the empirical survival functions of each country colored according to the final clustering and the marginal change points that characterizes each cluster.

Sampling from (8) is unfeasible due to the intractability of the normalization constant in each component of the mixture $\mathcal{L}(\rho \mid \boldsymbol{y}_i),\ i = 1, \ldots, n$. Normalising each component requires the computation of $\sum_{r=1}^{2^{T-1}} \mathcal{L}(\tilde{\rho}_r \mid \boldsymbol{y}_i)$ for each observation y_i, where $\{\tilde{\rho}_1, \ldots \tilde{\rho}_{2^{T-1}}\}$ are possible orders of T elements. Since the number of elements explodes as far as T increases, evaluating the normalization constants becomes soon unfeasible. We then proceed by pre-computing these constants using an importance sampling strategy: the importance distribution follows a binomial-multinomial law, for which we first generate the number of blocks k from a binomial distribution and then the frequencies from a multinomial distribution of dimension k. Then, we proceed with a standard impor-

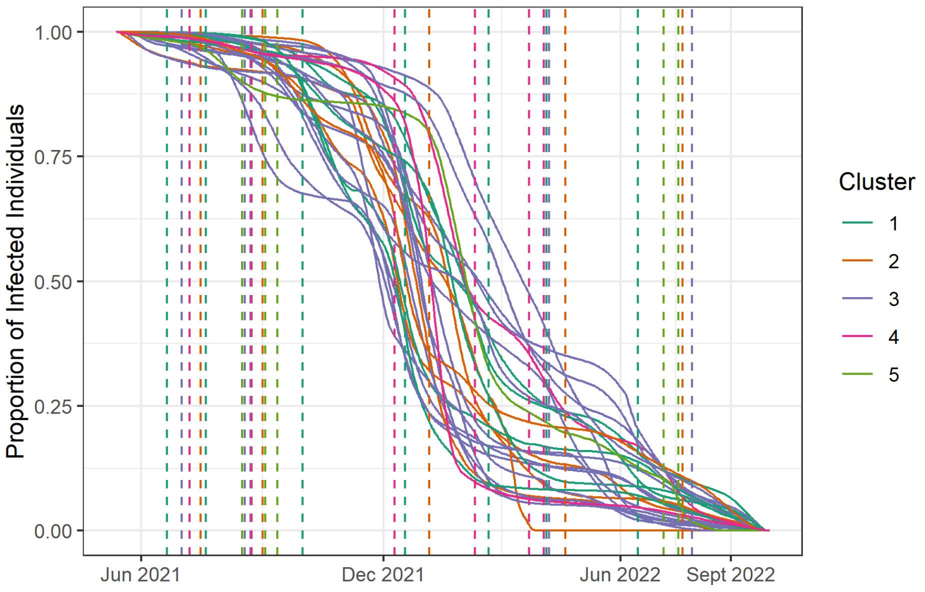

Figure 3: COVID-19 empirical survival functions of the EU countries. Colors represent the group in the final clustering and the dashed lines the marginal change points of each cluster.

tance reweighting approach. Algorithm 1 shows an implementation of such strategy for a single normalization constant.

Algorithm 1: Computation of the ith normalization constant of (8)

input number of sampled orders B for the importance sampling (accuracy) and success probability $p \in (0,1)$ of the binomial distribution.

for $r \in 1, \dots, \mathrm{B}$ **do**

a) **sample** $k \sim \text{Binomial}(T-1,\, p)$.

b) **sample** $\left(|B_1^{(r)}|, \dots, |B_k^{(r)}|\right)^\top \sim Multinom(T,\, (1/k, \dots, 1/k)^\top)$ and set $\rho_r^\dagger = \{B_1^{(r)}, \dots, B_k^{(r)}\}$.

d) **compute** $\phi(\rho_r^\dagger) = \binom{T}{k} p^k\, (1-p)^{T-k}\, T! \prod_{i=1}^{k} \frac{1}{\left(k^{|B_i^{(r)}|}\right)\left(|B_i^{(r)}|!\right)}$.

compute the approximation of $\sum_{r=1}^{2^{T-1}} \mathcal{L}(\tilde{\rho}_r \mid \boldsymbol{y}_i) \simeq \frac{2^{T-1}}{\mathrm{B}} \sum_{r=1}^{\mathrm{B}} \frac{1}{\phi(\rho_r^\dagger)} \mathcal{L}(\rho_r^\dagger \mid \boldsymbol{y}_i)$.

end

Finally, Algorithm 2 shows the pseudo-code for producing a sample of infection times in a fixed time window, bounded by the last observational time T. Here we consider an algorithm that works with a functional infection rate $\beta(u)$, which in our case is obtained as step function starting from a vector of local infection rates $\boldsymbol{\beta}$.

Algorithm 2: Doob-Gillespie algorithm to sample infection times

input a functional $\beta(u)$ of time-varying infection rates and a value ξ for the recovery rate, starting values for S_0, I_0 and R_0.

denote with (S_t, I_t, R_t) the vectors of susceptible, infected and recovered individuals at time t.

set $t = 0$ and $S_t = S_0$, $I_t = I_0$, $R_t = R_0$.

while $t < T$, $I_t > 0$ *and* $S_t > 0$ **do**

a) **sample** E_1 from $P(E_1 > e_1) = \exp\left(-\frac{S_t I_t}{S_0}\int_t^{t+e_1}\beta(u)du\right)$ and $E_2 \sim \text{Exp}(\xi I_t)$.

b) **set** $t^* = \min(E_1, E_2)$.

- **if** $t^* = E_1$, then set $S_t = S_t - 1$, $I_t = I_t + 1$, $R_t = R_t$, and $\delta = 1$.
- **else if** $t^* \neq E_1$, then set $S_t = S_t$, $I_t = I_t - 1$, $R_t = R_t + 1$, and $\delta = 0$.

c) Set $t = t + t^*$. If $\delta = 1$ **then** save t as new infection time.

end

i	$\{\beta_1^*, \beta_2^*\}$	I_0	$\{\lvert A_{i,1}\rvert, \lvert A_{i,2}\rvert\}$
1	{0.211, 0.55}	23/S0	{120, 80}
2	{0.221, 0.50}	23/S0	
3	{0.218, 0.54}	21/S0	
4	{0.225, 0.51}	20/S0	
5	{0.213, 0.52}	24/S0	{70, 130}
6	{0.213, 0.51}	23/S0	
7	{0.193, 0.57}	22/S0	
8	{0.195, 0.54}	21/S0	{30, 170}
9	{0.191, 0.53}	20/S0	
10	{0.189, 0.51}	24/S0	

Table 1: Parameters of the data generating processes for the epidemiological synthetic study. Left to right: observation index, different local infection rates for each series, starting infection rate and true latent order shared among observations in the same cluster.

References

Sonia Jain and Radford M Neal. A split-merge markov chain monte carlo procedure for the dirichlet process mixture model. *Journal of Computational and Graphical Statistics*, 13(1):158–182, 2004.

Sonia Jain and Radford M. Neal. Splitting and merging components of a nonconjugate Dirichlet process mixture model. *Bayesian Analysis*, 2(3):445 – 472, 2007.

Iosif Ilyich Gikhman and Anatoli Vladimirovich Skorokhod. *The Theory of Stochastic Processes II.* Springer Berlin Heidelberg, 2004.

David Applebaum. *Semigroups of Linear Operators: With Applications to Analysis, Probability and Physics.* Cambridge University Press, 07 2019.

Klaus-Jochen Engel and Rainer Nagel. *One-Parameter Semigroups for Linear Evolution Equations.* Springer-Verlag, 2000.

Håkan Andersson and Tom Britton. *Stochastic Epidemic Models and Their Statistical Analysis.* Springer New York, 2000.

Wasiur R. KhudaBukhsh, Boseung Choi, Eben Kenah, and Grzegorz A. Rempała. Survival dynamical systems: individual-level survival analysis from population-level epidemic models. *Interface Focus*, 10(1):20190048, 2020.

Francesco Di Lauro, Wasiur R. KhudaBukhsh, István Z. Kiss, Eben Kenah, Max Jensen, and Grzegorz A. Rempała. Dynamic survival analysis for non-markovian epidemic models. *Journal of The Royal Society Interface*, 19(191):20220124, 2022.

Wasiur R. KhudaBukhsh and Grzegorz A. Rempała. How to correctly fit an sir model to data from an seir model? *Mathematical Biosciences*, 375:109265, 2024.
